# Extension of *O*-Linked Mannosylation in the Golgi Apparatus Is Critical for Cell Wall Integrity Signaling and Interaction with Host Cells in Cryptococcus neoformans Pathogenesis

**DOI:** 10.1128/mbio.02112-22

**Published:** 2022-11-21

**Authors:** Eun Jung Thak, Ye Ji Son, Dong-Jik Lee, Hyunah Kim, Jung Ho Kim, Su-Bin Lee, Yu-Byeong Jang, Yong-Sun Bahn, Connie B. Nichols, J. Andrew Alspaugh, Hyun Ah Kang

**Affiliations:** a Department of Life Science, Chung-Ang University, Seoul, Republic of Korea; b Department of Biotechnology, College of Life Science of Biotechnology, Yonsei University, Seoul, Republic of Korea; c Department of Medicine, Duke University School of Medicine, Durham, North Carolina, USA; University of Texas Health Science Center

**Keywords:** *Cryptococcus neoformans*, protein *O*-mannosylation, *KTR3*, *CAP6*, fungal pathogenesis

## Abstract

The human-pathogenic yeast Cryptococcus neoformans assembles two types of *O*-linked glycans on its proteins. In this study, we identified and functionally characterized the C. neoformans *CAP6* gene, encoding an α1,3-mannosyltransferase responsible for the second mannose addition to minor *O*-glycans containing xylose in the Golgi apparatus. Two cell surface sensor proteins, Wml1 (WSC/Mid2-like) and Wml2, were found to be independent substrates of Cap6-mediated minor or Ktr3-mediated major *O*-mannosylation, respectively. The double deletion of *KTR3* and *CAP6* (*ktr3*Δ *cap6*Δ) completely blocked the mannose addition at the second position of *O*-glycans, resulting in the accumulation of proteins with *O*-glycans carrying only a single mannose. Tunicamycin (TM)-induced phosphorylation of the Mpk1 mitogen-activated protein kinase (MAPK) was greatly decreased in both *ktr3*Δ *cap6*Δ and *wml1*Δ *wml2*Δ strains. Transcriptome profiling of the *ktr3*Δ *cap6*Δ strain upon TM treatment revealed decreased expression of genes involved in the Mpk1-dependent cell wall integrity (CWI) pathway. Consistent with its defective growth under several stress conditions, the *ktr3*Δ *cap6*Δ strain was avirulent in a mouse model of cryptococcosis. Associated with this virulence defect, the *ktr3*Δ *cap6*Δ strain showed decreased adhesion to lung epithelial cells, decreased proliferation within macrophages, and reduced transcytosis of the blood-brain barrier (BBB). Notably, the *ktr3*Δ *cap6*Δ strain showed reduced induction of the host immune response and defective trafficking of ergosterol, an immunoreactive fungal molecule. In conclusion, *O*-glycan extension in the Golgi apparatus plays critical roles in various pathobiological processes, such as CWI signaling and stress resistance and interaction with host cells in C. neoformans.

## INTRODUCTION

Protein *O*-mannosylation, a type of posttranslational modification, is evolutionarily conserved from bacteria to humans with structural variation (1, 2). Protein *O*-mannosyltransferases (PMTs) initiate *O*-mannosylation in the endoplasmic reticulum (ER) by transferring mannose to serine or threonine residues of target proteins, a highly conserved aspect of protein glycosylation in eukaryotes (3, 4). However, the processing of *O*-mannosylation in the Golgi apparatus shows several species-specific features. In yeast and filamentous fungi, the α1,2-linked mannotriose (Manα1-2Manα1-2Manα1-Ser/Thr) is the most common core form, which is further extended in the Golgi apparatus by specific mannosyltransferases, generating short oligomannose chains with diverse structures depending on the species (5–8). In mammals, a variety of monosaccharides besides mannose can be *O*-linked to proteins, such as *N*-acetylgalactosamine, galactose, or glucose, followed by the addition of other sugars (9).

Protein *O*-mannosylation has critical biological roles, including directing cell morphology, protein stability, protein secretion, protein degradation, and cell wall integrity (CWI), in yeast and filamentous fungi (10–12). Involvement of *O*-mannosylation in virulence and development has been also reported for several fungal pathogens (13). In the human-pathogenic fungus Candida albicans, Pmt4-mediated *O*-mannosylation has roles in environment-specific morphogenetic signaling and full virulence (14). The disruption of C. albicans *MNT1* and *MNT2*, encoding partially redundant α-1,2-mannosyltransferases that catalyze the addition of the second and third mannose residues of *O*-glycans in the Golgi apparatus, results in altered cell morphology, hypersensitivity to cell wall-perturbing agents, and attenuated virulence in a murine model of systemic infection (15). In Aspergillus fumigatus, the *MNT1* deletion mutant strain shows reduced cell wall thickness, cell growth, conidium formation, and attenuated virulence (16). In the dimorphic fungal pathogen Histoplasma capsulatum, the *pmt1*Δ and *pmt2*Δ null mutants exhibit attenuated virulence because protein *O*-mannosylation is required for its thermotolerance at mammalian body temperature (17). Pleiotropic roles for the *O*-mannosyltransferase MoPmt4 were recently reported in the development and pathogenicity of the plant pathogen Magnaporthe oryzae (18). Additionally, *pmt*Δ mutants display impaired growth, reduced conidiation, defective cell walls, enhanced endoplasmic reticulum (ER) stress, and attenuated virulence in Fusarium oxysporum f. sp. *cucumerinum* (19).

Glycoproteins on the cell surface of fungal pathogens could contribute to interaction with host cells, and these interactions might be affected by altered glycan structures (20). In C. albicans, *pmt1*Δ and *mnt1*Δ *mnt2*Δ mutants are defective in their ability to induce host proinflammatory cytokines such as granulocyte colony-stimulating factor (G-CSF), granulocyte-macrophage colony-stimulating factor (GM-CSF), and interleukin 6 (IL-6). Moreover, these mutants show reduced ability to damage oral epithelial cells (21). Cell wall remodeling resulting in exposed glycosylated proteins is required for C. albicans*-*induced pyroptosis, a form of necrotic cell death mediated by inflammasomes (22, 23). Interestingly, *O*-mannosylated proteins, such as mannoprotein Dan1 acting as an ergosterol receptor, have been proposed to affect pyroptosis by altering ergosterol localization. Pmt4-mediated *O*-mannosylation has been shown to be especially required for C. albicans-induced pyroptosis (24).

Cryptococcus neoformans is an opportunistic fungal pathogen that causes life-threatening meningoencephalitis in immunocompromised hosts after crossing the blood-brain barrier (BBB). In pathogenic Cryptococcus species, several extracellular factors contribute to virulence, including the polysaccharide capsule, melanin pigment, and various hydrolytic enzymes (25). In addition, many secreted mannoproteins are key factors of C. neoformans pathogenicity (26, 27) and evoke specific immune responses (28). In C. neoformans, the PMT family, composed of three members, Pmt1, Pmt2, and Pmt4, was previously reported to initiate protein *O*-mannosylation. *PMT2* is an essential gene, and the *pmt1*Δ and *pmt4*Δ mutants show attenuated virulence in a murine inhalation model of cryptococcosis (10, 29). The presence of a xylose-phosphate-mannose linkage, mediated by Xpt1, was reported in C. neoformans *O*-linked glycans (30). Subsequently, systematic analysis of the *O*-glycan structure indicated that C. neoformans has two *O*-mannosylation pathways, a major pathway generating short mannosylated glycans without xylose modification (Man_1_–Man_4_) and a minor pathway with xylose addition (Xyl_1_Man_2_–Xyl_1_Man_4_) (31).

In this study, we report that C. neoformans Cap6 is responsible for the second mannose addition in α1,3-linkage to the minor *O*-glycans in the Golgi apparatus. Notably, two novel cell surface sensor proteins, Wml1 and Wml2, are identified as independent substrates of Cap6-mediated minor and Ktr3-mediated major *O*-mannosylation, respectively. By systematic analysis of the *ktr3*Δ *cap6*Δ double mutant, we demonstrate the critical roles of the *O*-glycan extension in activating CWI signaling pathways and in mediating host cell interaction during sequential steps of the infection process. We further present data supporting the requirement of *O*-glycan extension for the host immune response, partly by involvement in ergosterol trafficking.

## RESULTS

### Cap6 adds the second mannose residue for xylose addition in minor *O*-linked glycans.

Cryptococcus neoformans has two *O*-mannosylation pathways, the major pathway that mediates *O*-mannosyl additions that do not contain xylose and the minor pathway that mediates additions containing xylose (31). Ktr3 (α1,2-mannosyltransferase) is responsible for the addition of the second mannose residue in the major *O*-glycan synthesis pathway. However, the presence of xylose-containing *O*-glycans in the *ktr3*Δ mutant implies that extension of minor *O*-glycans in C. neoformans occurs by the action of another specific mannosyltransferase, independent of Ktr3. We hypothesized that the mannose residue at the second position of minor *O*-glycans, at which xylose is attached (31), should have an α1,3-linkage considering that a xylose residue is attached to the mannose backbone that has α1,3-linkage in C. neoformans capsules (32, 33) and *N*-glycans (34). To confirm this possibility, we collected and analyzed the M2 peak from the C. neoformans *ktr3*Δ strain (see Fig. S1A in the supplemental material). Comparison of the high-performance liquid chromatography (HPLC) elution profile with standard α1,2-mannobiose and α1,3-mannobiose indicated that the second mannose residue of C. neoformans minor *O*-glycans is added in an α1,3-linkage to the first mannose residue (Fig. S1A), in contrast to the α1,2-linkage of the second mannose residue in major *O*-glycans. To define the α1,3-mannosyltransferase that adds the second mannose residue to minor *O*-glycans in C. neoformans, we selected two proteins, Cap6 (CNAG_06016) and Cmt1 (CNAG_03158), which were previously identified based on their homology to Cap59, an α1,3-mannosyltransferase involved in capsule synthesis (35, 36). Multiple sequence alignment of Cap59 homologs revealed that C. neoformans Cap59, Cap6, and Cmt1 have a DXD (aspartate-any residue-aspartate) motif, essential for enzymatic activity and commonly found in glycosyltransferases (Fig. S1B). Cmt1 was suggested to be involved in the synthesis of the capsule polymer glucuronoxylomannan (GXM), but deletion of *CMT1* does not affect capsule formation (35). Cap6 is homologous to both Cmt1 and Cap59 (36). However, a previous deletion study similarly reported that Cap6 is not involved in capsule formation (37), leaving open questions on the physiological role of Cap6.

10.1128/mbio.02112-22.3 FIG S1 Identification and bioinformatics analysis of Cap6 as α1,3-mannosyltransferase involved in the minor *O*-mannosylation of C. neoformans. (A) Comparative analysis of HPLC elution patterns of C. neoformans *ktr3*Δ M2 with the standard α1,2- and α1,3-mannobioses (α1,2M and α1,3M). HPLC analysis was conducted using a Asahipak NH2P-50 4E column (0.46 by 25 cm, 5 μm; Shodex). (B) Multiple sequence alignment of Cap6 homologs generated using ClustalW 1.8. Identical residues and conservative amino acid substitutions are indicated by black and gray shading, respectively. The aspartate-any residue-aspartate (DXD) motif is boxed. Download FIG S1, TIF file, 1.6 MB. Copyright © 2022 Thak et al. 2022 Thak et al. https://creativecommons.org/licenses/by/4.0/ This content is distributed under the terms of the Creative Commons Attribution 4.0 International license.

**FIG 1:**
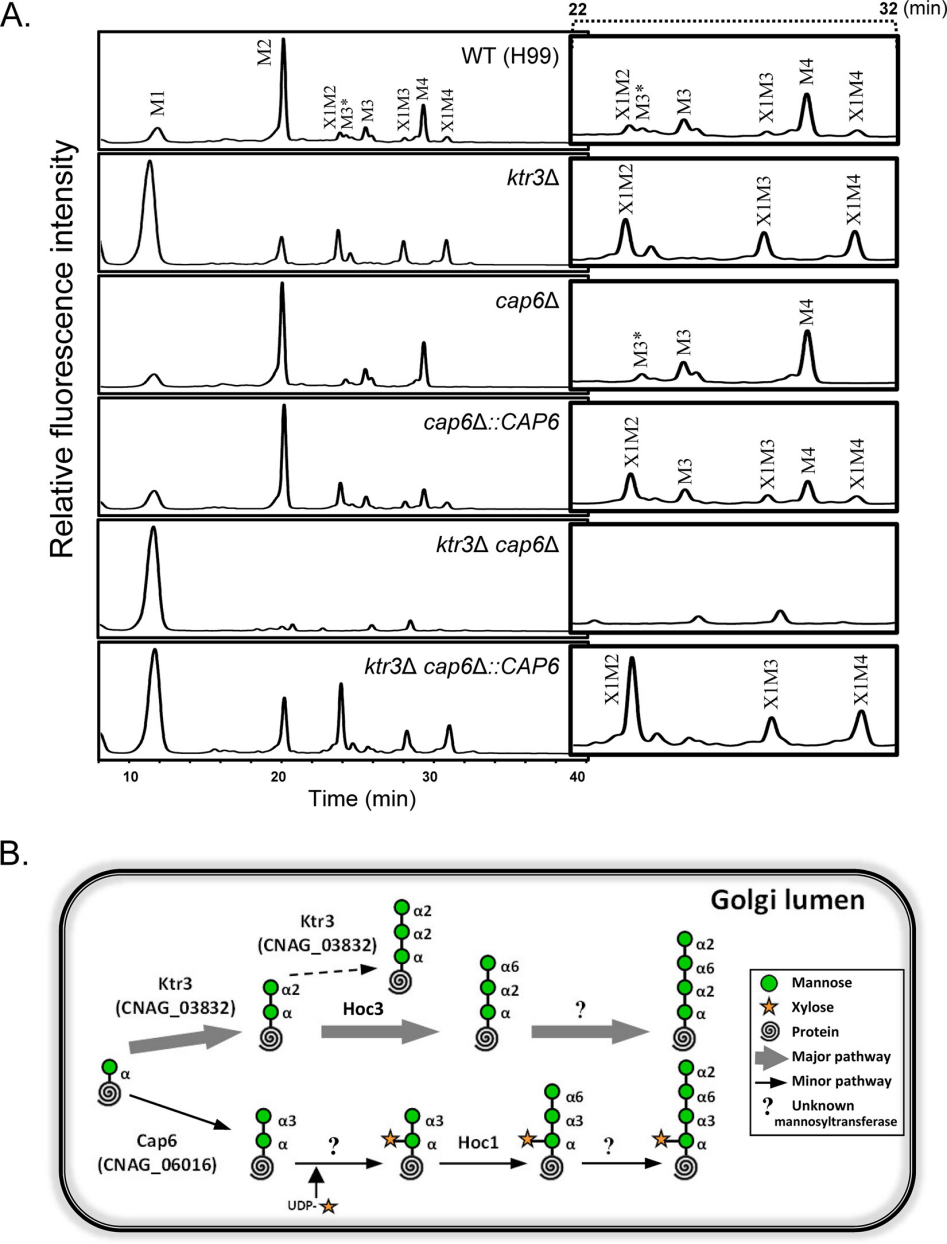
Functional analysis of Cap6 as α1,3-linked mannosyltransferase responsible for biosynthesis of minor *O*-linked glycans. (A) HPLC profiles of *O*-glycans of cell wall mannoproteins (cwMPs) from WT, *ktr3*Δ, *cap6*Δ, *cap6*Δ::*CAP6*, *ktr3*Δ *cap6*Δ, and *ktr3*Δ *cap6*Δ::*CAP6* strains. To distinguish small peaks more clearly, the HPLC profiles from 22 to 32 min in the *x* and *y* axes were enlarged and inserted. M, mannose; X, xylose; M3*, α1,2-mannotriose. (B) Schematic representation of the proposed *O*-glycosylation pathway in C. neoformans assigned with Cap6.

We disrupted the *CMT1* and *CAP6* genes (Fig. S2A) and analyzed the *O*-glycan profiles of the *cmt1*Δ and *cap6*Δ strains. Although no apparent differences were observed in the *O*-glycan profile of the *cmt1*Δ mutant compared with that of the wild-type (WT) strain (data not shown), the *cap6*Δ mutant showed reduced peaks corresponding to the minor X1M2 to X1M4 oligosaccharides by HPLC analysis. Reintroduction of the WT *CAP6* gene into the *cap6*Δ mutant restored the X1M2 to X1M4 peaks (Fig. 1A). The *O*-glycan profile of the *ktr3*Δ *cap6*Δ double mutant strain displayed only the M1 peak, in which the first mannose residue is added to serine or threonine of proteins by *PMT* gene families in the ER (Fig. 1A). Reintroduction of *CAP6* and *KTR3* into the *ktr3*Δ *cap6*Δ mutant (*ktr3*Δ *cap6*Δ::*CAP6* strain and *ktr3*Δ *cap6*Δ::*KTR3* strain) (Fig. S2B and C) restored the altered *O*-glycan profile to the almost identical profiles of the *ktr3*Δ and *cap6*Δ strains, respectively (Fig. 1A; Fig. S2D). These results demonstrate that Cap6 is responsible for the addition of the second α1,3-mannose residue in the biosynthetic pathway of minor *O*-glycans (Fig. 1B). This also strongly supports that the α1,3-linkage is an important determinant for xylose addition not only in capsules but also in glycans assembled on proteins.

10.1128/mbio.02112-22.4 FIG S2 Construction of *CAP6* disruption, complementation strains, and *in vitro* analysis of virulence-associated phenotypes. (A) Strategy for CNAG_06016 disruption in WT and *ktr3*Δ mutant background, respectively. (B) Strategy for *CAP6* complementation in *cap6*Δ and *ktr3*Δ *cap6*Δ mutant background, respectively. (C) Complementation of *KTR3* in *ktr3*Δ *cap6*Δ mutant strain using the safe haven strategy. Stable transformants were selected on YPD medium containing hygromycin B (150 μg/mL) and screened by PCR. (D) HPLC profiles of *O*-glycans of cwMPs from WT, *ktr3*Δ, *ktr3*Δ::*KTR3*, *cap6*Δ, *cap6*Δ::*CAP6*, *ktr3*Δ *cap6*Δ, *ktr3*Δ *cap6*Δ::*CAP6*, and *ktr3*Δ *cap6*Δ::*KTR3* strains. (E) Spotting analysis of growth of the C. neoformans WT, *ktr3*Δ, *cap6*Δ, *ktr3*Δ *cap6*Δ, *ktr3*Δ *cap6*Δ::*CAP6*, and *ktr3*Δ *cap6*Δ::*KTR3* strains under conditions of heat stress (37 or 39°C), ER stress (TM), and cell wall stress (CFW, SDS, and VAN). (F) Analysis of capsule formation. (G) Analysis of melanin synthesis. The *cac1*Δ strain was included as a control strain showing defective melanization on l-3,4-dihydroxyphenylalanine (l-DOPA) medium. Download FIG S2, TIF file, 2.9 MB. Copyright © 2022 Thak et al. 2022 Thak et al. https://creativecommons.org/licenses/by/4.0/ This content is distributed under the terms of the Creative Commons Attribution 4.0 International license.

### Ktr3 and Cap6 function independently in *O*-linked protein mannosylation of C. neoformans.

**FIG 2:**
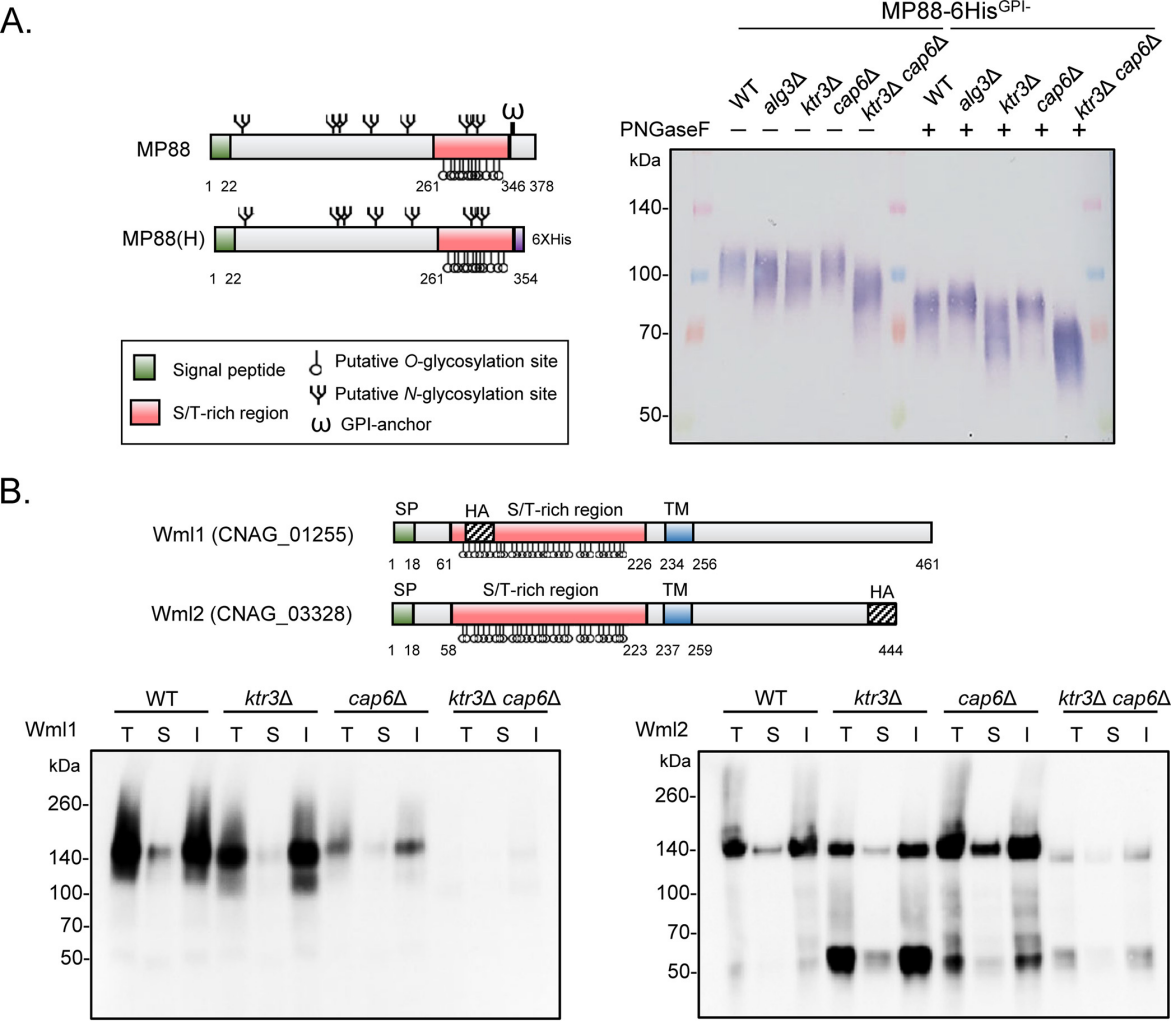
Glycosylation pattern analysis of C. neoformans *O*-glycan mutant strains. (A) Western blot of MP88 protein secreted from several *N*- or *O*-glycosylation mutants of C. neoformans, including *alg3*Δ, *ktr3*Δ, *cap6*Δ, and *ktr3*Δ *cap6*Δ mutants. Yeast cells were cultivated in YPD medium for 24 h and harvested, and the culture supernatants were TCA precipitated and subjected to Western blot analysis with anti-His antibody. (B) Western blot analysis of Wml1 and Wml2 in C. neoformans *O*-glycan-deficient mutant strains. Domain structure of putative cell wall stress sensors, Wml1 and Wml2. Circle symbol indicates *O*-glycosylation sites, predicted by YinOYang1.2. Subcellular fractionation was subjected to Western blot analysis with anti-HA antibody, as described in Text S1 in the supplemental material. T, total; S, soluble; I, insoluble; TM, transmembrane domain.

To investigate the effect of *KTR3* and *CAP6* deletions on protein *O*-mannosylation, we examined the glycosylation pattern of mannoprotein 88 (MP88), which was reported to stimulate T-cell responses and to be involved in the C. neoformans-epithelial lung cell adhesion (38). MP88 is predicted to contain 7 *N*-glycosylation sites and 41 *O*-glycosylation sites. To analyze the glycosylation patterns of MP88, His-tagged and glycosylphosphatidylinositol (GPI)-anchorless MP88 was expressed in various glycosylation mutant strains, including *alg3*Δ, *ktr3*Δ, *cap6*Δ, and *ktr3*Δ *cap6*Δ strains (Fig. 2A). The migration of MP88 protein was altered in the *alg3*Δ mutant, which has a defect in the biosynthesis of core *N*-linked glycans (39), indicating that MP88 protein is subjected to *N*-glycosylation. The MP88 protein did not show a detectable change in apparent molecular weight (MW) in the *cap6*Δ mutant but displayed a noticeable decrease in MW with more smeared bands in the *ktr3*Δ mutant strain. The MP88 protein band of the *ktr3*Δ *cap6*Δ mutant migrated substantially faster than that of the WT and *ktr3*Δ strains. This strongly indicates that MP88 is highly modified by *O*-glycosylation by Ktr3, along with an additional contribution of Cap6, such that the extension of *O*-mannosylation is completely defective in the *ktr3*Δ *cap6*Δ strain.

10.1128/mbio.02112-22.1 TEXT S1 Supplemental methods and references. Download Text S1, DOCX file, 0.05 MB. Copyright © 2022 Thak et al. 2022 Thak et al. https://creativecommons.org/licenses/by/4.0/ This content is distributed under the terms of the Creative Commons Attribution 4.0 International license.

To identify other protein targets to be highly *O*-mannosylated, we explored glycosylation patterns of the C. neoformans homologs of two Saccharomyces cerevisiae cell surface sensor proteins (Fig. 2B). The WSC family (Wsc1 to -4 proteins) and the Mid2p-Mtl1p pair sense environmental stresses in S. cerevisiae. These proteins are highly *O*-mannosylated within Ser/Thr-rich domains and are involved in the MAPK signaling pathway, activating cell wall synthesis to maintain cell wall integrity (40). These putative cell wall stress sensors were identified in the C. neoformans proteome through four sequential *in silico* analyses: (i) proteins containing a region of 40 amino acids in which the Ser/Thr content was ≥40% (958 proteins); (ii) proteins containing a single-pass transmembrane domain at the C-terminus (80 proteins); (iii) the presence of a signal peptide at the N-terminus without a GPI anchor domain (12 proteins); and (iv) the presence of putative *O*-glycosylation sites (2 proteins) (Fig. S3A). This analysis resulted in two putative cell wall sensors of C. neoformans, CNAG_01255 and CNAG_03328, each with a highly Ser/Thr-rich region subject to *O*-glycosylation. CNAG_01255 and CNAG_03328 are predicted to have 83 sites and 79 sites, respectively. Therefore, we named CNAG_01255 Wml1 (Wsc-Mid2-like 1) and CNAG_03328 Wml2 (Wsc-Mid2-like 2). The null mutants of cell wall stress sensors, *wml1*Δ, *wml2*Δ, and *wml1*Δ *wml2*Δ mutants, were constructed (Fig. S4) and analyzed for their growth phenotypes (Fig. S5A). Although the *wml1*Δ, *wml2*Δ, and *wml1*Δ *wml2*Δ mutant strains exhibited no detectable growth defects under other stress conditions, *wml2*Δ and *wml1*Δ *wml2*Δ mutant strains showed significantly increased sensitivity to salt stress caused by the presence of 1 M NaCl (Fig. S5A). The salt sensitivity to 1 M NaCl was also restored by 1 M sorbitol (Fig. S5B). The *wml1*Δ mutant has a very subtle defect in growth on salt, but the *wml1*Δ *wml2*Δ double mutant has a synthetic effect on growth on salt, indicating that both Wml1 and Wml2 play overlapping and partially redundant roles in the salt stress response.

10.1128/mbio.02112-22.5 FIG S3 Bioinformatics analysis of cell wall stress sensors in various yeast species. (A) Flow chart to screen putative cell wall stress sensors through *in silico* analysis. (B) Domain structure of cell wall stress sensors. S. cerevisiae (*Sc*Wsc1, NP_014650.1; *Sc*Wsc2, NP_014116.1; *Sc*Wsc3, NP_014536.1; *Sc*Mid2, NP_013436.1; *Sc*Mtl1, NP_011537.1), C. albicans (*Ca*Wsc1, XP_723083.2; *Ca*Wsc2, XP_719101.2), S. pombe (*Sp*Wsc1, NP_595526; *Sp*Mtl2, NP_594395.1). (C) Phylogenetic analysis of cell wall stress sensors. The phylogenetic tree was constructed by the maximum likelihood methods in MEGA X software, and a bootstrap analysis with 100 random resamplings was used. (D) Sequence identities and similarities between cell wall stress sensor homologs in various yeast species. Download FIG S3, TIF file, 2.0 MB. Copyright © 2022 Thak et al. 2022 Thak et al. https://creativecommons.org/licenses/by/4.0/ This content is distributed under the terms of the Creative Commons Attribution 4.0 International license.

10.1128/mbio.02112-22.6 FIG S4 Disruption of putative cell wall stress sensors, CNAG_01255 and CNAG_03328, and construction of a complementation strain. Strategy for CNAG_01255 or CNAG_03328 disruption using the nourseothricin acetyltransferase (NAT) split marker. Stable transformants were selected on YPD medium containing nourseothricin (100 μg/mL) and were screened by PCR. Strategy for CNAG_01255 and CNAG_03328 double disruption using the neomycin (NEO) split marker. Stable transformants were selected on YPD medium containing G418 (200 μg/mL) and were screened by PCR. Scheme of construction of an CNAG_01255Δ CNAG_03328Δ::CNAG_03328 complementation strain. Download FIG S4, TIF file, 1.1 MB. Copyright © 2022 Thak et al. 2022 Thak et al. https://creativecommons.org/licenses/by/4.0/ This content is distributed under the terms of the Creative Commons Attribution 4.0 International license.

10.1128/mbio.02112-22.7 FIG S5 Growth analysis of C. neoformans *wml1*Δ, *wml2*Δ, and *wml1*Δ *wml2*Δ cell wall sensor mutants under various stress conditions. (A) Spotting analysis of growth of C. neoformans *wml1*Δ, *wml2*Δ, *wml1*Δ *wml2*Δ, and *wml1*Δ *wml2*Δ::*WML2* putative cell wall sensor mutant strains under heat stress (37°C, 39°C), ER stress (TM, tunicamycin; DTT, dithiothreitol), cell wall stress (SDS, caffeine), osmotic stress (NaCl, sorbitol) and pH stress (pH 4, pH 8). (B) Spotting analysis of growth of *wml1*Δ, *wml2*Δ, *wml1*Δ *wml2*Δ, and *wml1*Δ *wml2*Δ::*WML2* strains in the presence of NaCl with and without 1 M sorbitol supplementation. (C) Cell wall stress sensor mutant strains, *O*-glycan *ktr3*Δ *cap6*Δ mutant strain, and *hog1*Δ and *msb2*Δ mutant strains were tested in osmotic stress medium (1 M NaCl) containing the glucose or no-glucose conditions. The representative image shows the data at day 3. (D) Dephosphorylation of Hog1 induced by 1 M NaCl in WT, *O*-glycosylation mutants, and cell wall stress sensor mutants. C. neoformans cells were exposed to 1 M NaCl in YPD medium for the indicated time, and total proteins were extracted for Western blot analysis, as described in Text S1 in the supplemental material. Dual phosphorylation of Hog1 (T180/T182) was detected by phospho-p38-MAPK antibody. The same blots were stripped and reprobed with anticryptococcal polyclonal Hog1 antibody as a loading control. Download FIG S5, TIF file, 1.6 MB. Copyright © 2022 Thak et al. 2022 Thak et al. https://creativecommons.org/licenses/by/4.0/ This content is distributed under the terms of the Creative Commons Attribution 4.0 International license.

In subcellar fractionation experiments, hemagglutinin (HA)-tagged Wml1 and Wml2 were mainly detected in the insoluble fraction (cell wall/membrane) (Fig. 2B). The Wml1 and Wml2 proteins were expressed as forms much larger (≥140 kDa) than the predicted size (approximately 56 kDa) in the WT strain, suggesting extensive posttranslational modification. Moreover, the expression levels of Wml1 and Wml2 were different in the *O*-glycan-deficient *ktr3*Δ, *cap6*Δ, and *ktr3*Δ *cap6*Δ strains. The expression levels of Wml1 were decreased in the *O*-glycan-deficient strains, with a more dramatic decrease in the *cap6*Δ mutant than the *ktr3*Δ mutant, indicating that Wml1 is mainly subjected to minor *O*-mannosylation. In the *ktr3*Δ *cap6*Δ mutant strain, we could not detect the protein bands corresponding to Wml1 protein. In contrast, Wml2 proteins with lower MW accumulated more in the *ktr3*Δ mutant than in the *cap6*Δ mutant, suggesting that this protein is largely glycosylated by major *O*-mannosylation via Ktr3. Further decreased levels of Wml2 proteins in both high- and low-MW forms were detected in the *ktr3*Δ *cap6*Δ strain. This clearly indicates that *O*-linked glycans of Wml1 and Wml2 were differently modified by minor and major *O*-linked mannosylation, respectively. The signals for the protein bands of Wml1 and Wml2 were both greatly decreased in the *ktr3*Δ *cap6*Δ mutant (Fig. 2B), supporting that C. neoformans Wml1 and Wml2 are highly *O*-glycosylated at the Ser/Thr region, which is required for their stability.

### Extended mannosylation of *O*-linked glycans is essential for the adaptation responses to various external stress.

**FIG 3:**
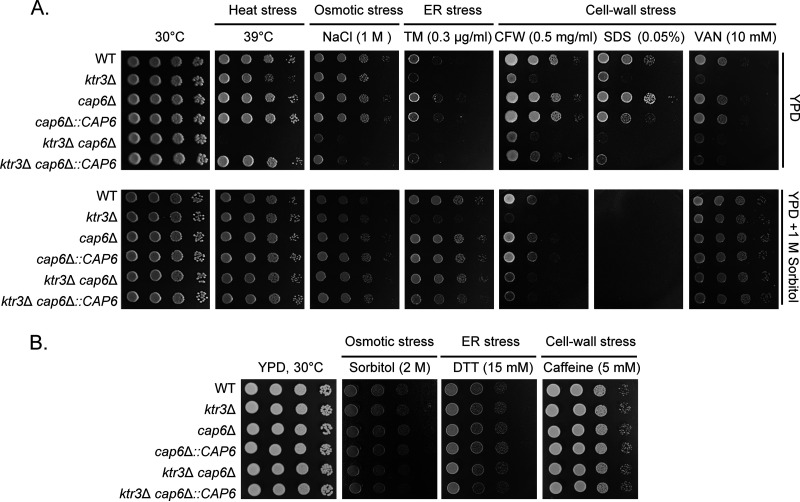
Growth phenotypes of C. neoformans *O*-glycan mutant strains. (A) Growth analysis of C. neoformans *O*-glycan mutant strains with or without 1 M sorbitol under different conditions. Serially diluted cells were spotted on YPD plates with or without 1 M sorbitol under different conditions, including heat stress (39°C), ER stress (TM), cell wall stress (CFW, calcofluor white; SDS, sodium dodecyl sulfate; VAN, vanadate), and osmotic stress (NaCl). (B) Spotting analysis of growth of C. neoformans *O*-glycan mutant strains in the presence of DTT, caffeine, and sorbitol.

To assess the physiological impact of a complete block of extension of *O*-mannosylation, the WT and *ktr3*Δ, *cap6*Δ, and *ktr3*Δ *cap6*Δ mutant strains were cultivated on yeast extract-peptone-dextrose (YPD) plates under various stress conditions, including heat, osmotic, ER, and cell wall stresses (Fig. 3A and B). The *ktr3*Δ mutant, defective in major *O*-glycan biosynthesis, displayed increased sensitivity to high temperature (39°C), NaCl, tunicamycin (TM), calcofluor white (CFW), sodium dodecyl sulfate (SDS), and vanadate (VAN). In contrast, the *cap6*Δ mutant, defective in minor *O*-glycan biosynthesis, did not display any detectable growth alteration under the tested stress conditions except SDS treatment. Interestingly, the *cap6*Δ mutant appeared to be slightly more resistant to SDS. The *ktr3*Δ *cap6*Δ double mutant exhibited increased sensitivity to high temperature, NaCl, and SDS compared to the *ktr3*Δ single mutant (Fig. 3A). Reintroduction of *CAP6* into the *ktr3*Δ *cap6*Δ mutant restored the growth defects at high temperature and in the presence of SDS to levels similar to those of the *ktr3*Δ mutant (Fig. 3A). Reintroduction of *KTR3* into the double mutant complemented more fully the growth phenotypes to levels similar to those of the *cap6*Δ strain (Fig. S2E). The growth defects of the *ktr3*Δ *cap6*Δ mutant under stress conditions, except CFW treatment, were also almost completely suppressed by the addition of 1 M sorbitol as an osmotic stabilizer, suggesting that the stress sensitivity of *O*-mannosylation mutants is mainly caused by defective cell wall integrity. In contrast, the *ktr3*Δ single mutant and even the *ktr3*Δ *cap6*Δ double mutant did not show increased sensitivity to 2 M sorbitol, dithiothreitol (DTT), or caffeine compared to the WT strain (Fig. 3B). Capsule formation and melanin synthesis in the *O*-mannosylation mutants did not differ significantly from those in the WT strain (Fig. S2F and G). These results suggest that the extended structure of the *O*-glycans in the Golgi apparatus, including major and minor *O*-glycans, is critical for the adaptative responses to several external stresses in C. neoformans but dispensable for capsule and melanin formation.

### *O*-Mannosylation plays a role in the Mpk1-cell wall integrity signaling pathway but not in the Hog1-salt stress signaling pathway.

**FIG 4:**
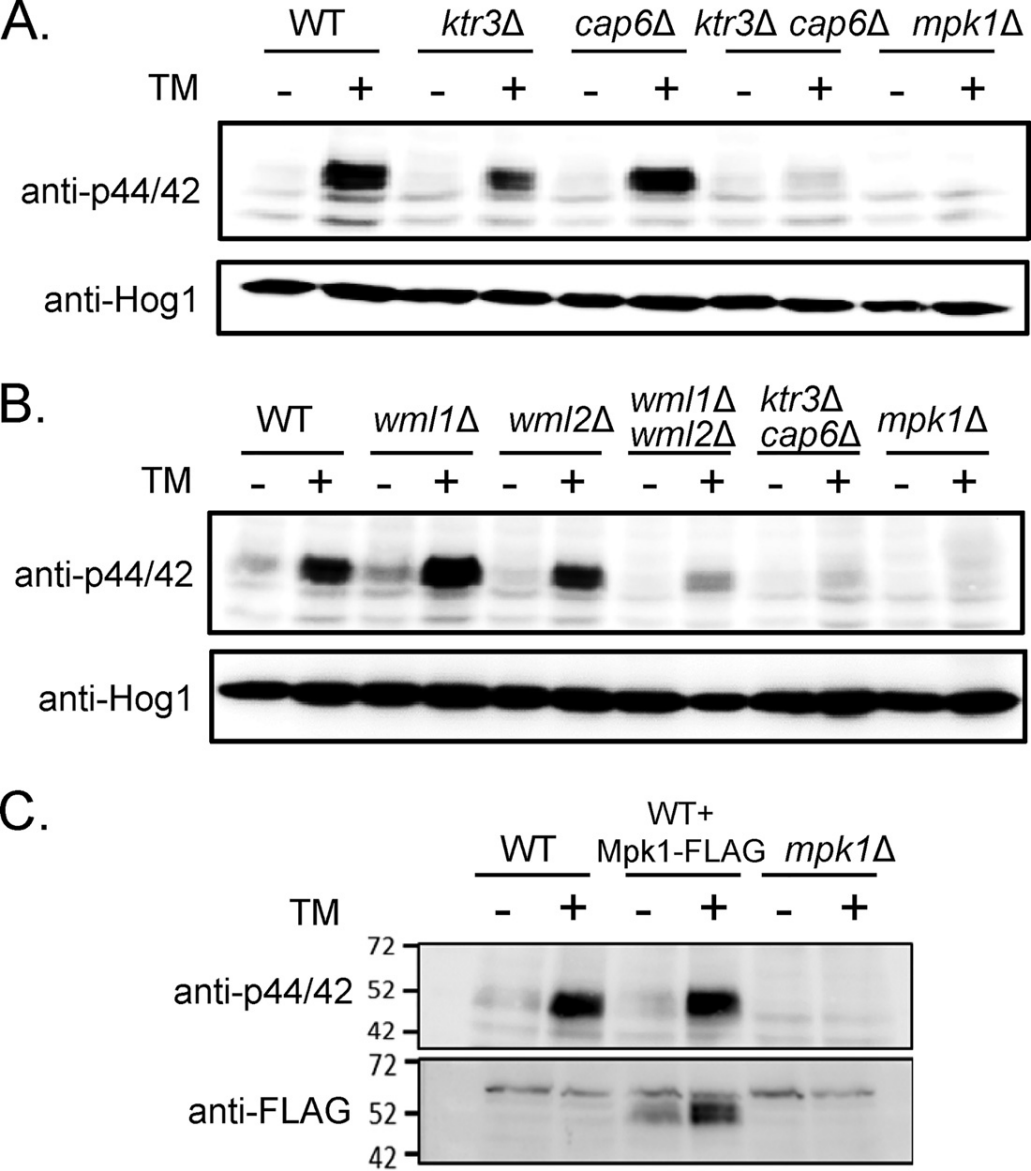
Analysis of MAPK-mediated stress response signaling activity. (A and B) Phosphorylation of Mpk1 induced by TM in the WT, *O*-glycosylation mutants (*ktr3*Δ, *cap6*Δ, and *ktr3*Δ *cap6*Δ mutants), and cell wall stress sensor mutants (*wml1*Δ, *wml2*Δ, and *wml1*Δ *wml2*Δ mutants). Phosphorylated Mpk1 (Mpk1-p) was detected with phospho-p44/42-MAPK antibody, and Hog1 protein was detected with anti-Hog1 as a loading control, as described in Text S1 in the supplemental material. (C) Expression level of Mpk1 protein induced under the TM treatment condition. The pattern of phosphorylated Mpk1 (Mpk1-p) was detected with phospho-p44/42-MAPK antibody and Mpk1 protein with anti-FLAG.

We examined whether truncated *O*-mannosylation would generate defects in C. neoformans Mpk1- and Hog1-mediated stress signaling pathways. TM treatment was previously shown to induce phosphorylation of the CWI-controlling Mpk1 MAP kinase in C. neoformans (41) (Fig. 4A). Although the levels of phosphorylated Mpk1 decreased moderately in the *ktr3*Δ mutant and had no detectable change in the *cap6*Δ mutant, they markedly decreased in the *ktr3*Δ *cap6*Δ mutant compared to the WT upon TM treatment (Fig. 4A). The levels of phosphorylated Mpk1 were also markedly decreased in the *wml1*Δ *wml2*Δ double mutant compared to the WT upon TM treatment, even though there was no apparent decrease in Mpk1 phosphorylation in the *wml1*Δ or *wml2*Δ single mutant strains (Fig. 4B). Mpk1 total protein levels also increased upon TM treatment (Fig. 4C). Taken together, these results suggest that defective *O*-mannosylation and the loss of the putative cell wall stress sensors Wml1 and Wml2 are important components in the translation of cell stress, such as TM-mediated ER stress, to the activation of the Mpk1-mediated CWI signaling pathway.

Since the Hog1 MAPK plays a role in maintaining osmotic balance, particularly under carbon-starved conditions (42), we compared osmotic stress response phenotypes under both glucose-rich and -starved conditions. Remarkably, the salt sensitivity of the *wml1*Δ *wml2*Δ double mutant was more severe than that of the *ktr3*Δ *cap6*Δ mutant and the *msb2*Δ mutant strain, which has a null mutation of *MSB2* encoding a mucin-like transmembrane protein involved in osmosensing (43), indicating that the Wml proteins play an important role in salt stress response (Fig. S5C). Interestingly, the *wml1*Δ *wml2*Δ and *ktr3*Δ *cap6*Δ double mutant strains displayed similar levels of osmosensitivity under both glucose-rich and -starved conditions, indicating that *O*-glycans play important roles in maintaining osmotic balance, which might be operated in a Hog1-independent manner (Fig. S5C). To further assess whether the contribution of *O*-glycans to osmoadaptation is associated with Hog1, we analyzed the Hog1 phosphorylation patterns in WT and *O*-glycan mutant strains in response to 1 M NaCl (Fig. S5D). In the WT strain, phosphorylated Hog1 was gradually dephosphorylated upon exposure to 1 M NaCl, as previously reported (44). A similar pattern of Hog1 dephosphorylation was also observed in the *ktr3*Δ *cap6*Δ and *wml1*Δ *wml2*Δ double mutant strains under osmotic stress by 1 M NaCl. This indicates that *O*-glycosylation is not required for Hog1 activation in response to osmotic/salt stress.

### Transcriptome analysis reveals defective Mpk1-mediated stress response signaling pathway in *O*-glycan mutants.

**FIG 5:**
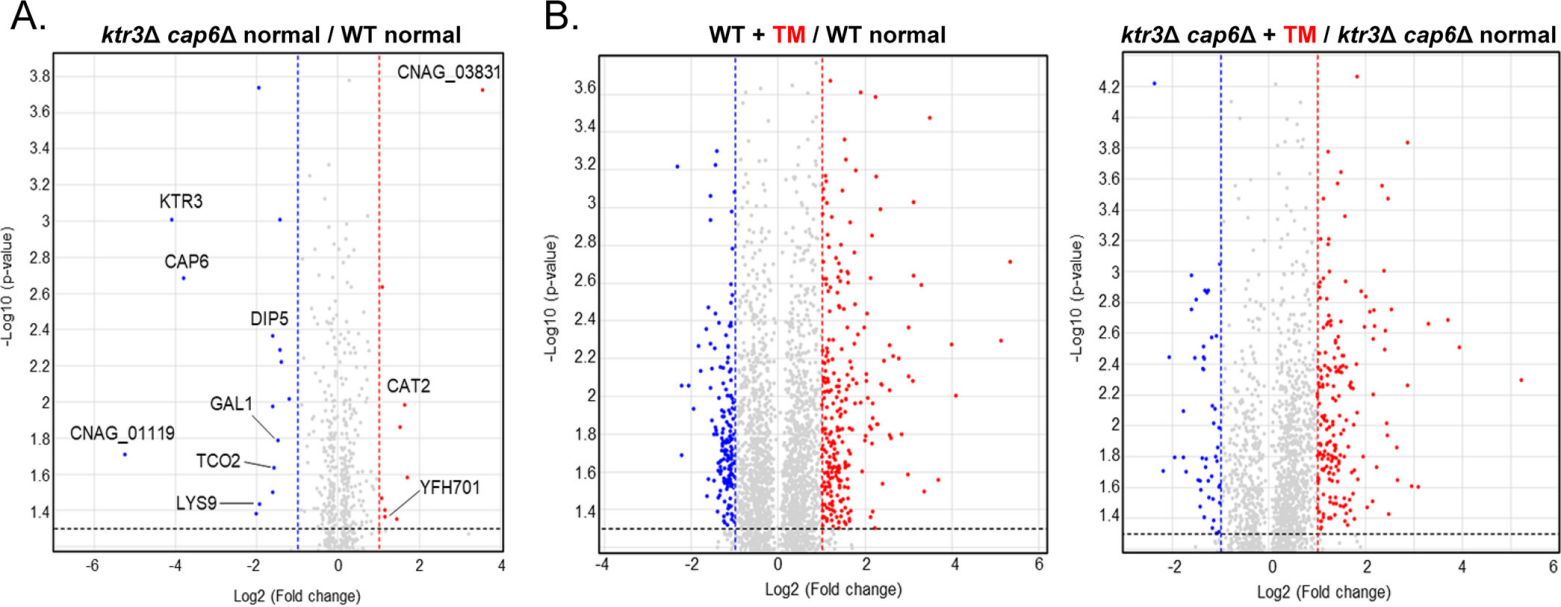
Comparative RNA-Seq analysis of the WT and *ktr3*Δ *cap6*Δ strains. (A) Volcano plot comparing log_2_ (2-fold change) values for the WT versus *ktr3*Δ *cap6*Δ strains under normal growth conditions. (B) Volcano plot comparing log_2_ (2-fold change) values for the WT versus *ktr3*Δ *cap6*Δ strains upon tunicamycin (TM) treatment. C. neoformans WT and mutant cells at an OD_600_ of 0.15 were cultured in 50 mL of YPD broth at 30°C in a shaking incubator until the OD_600_ reached 0.8, the early phase of exponential growth. The cells were then treated with 5 μg/mL TM and further incubated at 30°C in a shaking incubator for 1 h. Total RNA was extracted and subjected to RNA-Seq analysis as described in Text S1 in the supplemental material.

To elucidate regulatory networks with downstream components governed by *O*-mannosylation, we performed RNA sequencing (RNA-Seq)-based transcriptome analysis. The transcriptome profiles of the WT and *ktr3*Δ *cap6*Δ cells treated with TM (5 μg/mL) for 1 h were compared to those without TM treatment. First, we analyzed the genes that were differentially expressed more than 2-fold between the WT and *ktr3*Δ *cap6*Δ cells cultivated in YPD medium (Fig. 5A). Under this permissive growth condition, *CAT2* (catalase 2) and CNAG_03831 (unknown function) were highly upregulated in the *ktr3*Δ *cap6*Δ mutant compared to those in the WT strain, while the genes involved in transmembrane transport, such as CNAG_01119 encoding a POT family proton-dependent oligopeptide transporter, were significantly downregulated (Fig. 5A; Fig. S6A) in the *ktr3*Δ *cap6*Δ mutant compared to those in the WT.

10.1128/mbio.02112-22.8 FIG S6 Comparative transcriptome analysis of WT and *ktr3*Δ *cap6*Δ mutant strains. (A) Functional categories of upregulated or downregulated genes in the *ktr3*Δ *cap6*Δ strain compared to the WT strain under normal growth conditions. (B) Venn diagram of the genes upregulated or downregulated by more than a 2-fold change under TM-induced ER stress conditions relative to the normal condition. (C) DAVID analysis-based enrichment scores of gene ontology (GO) terms for genes upregulated (red) or downregulated (blue) in response to TM treatment. Download FIG S6, TIF file, 1.2 MB. Copyright © 2022 Thak et al. 2022 Thak et al. https://creativecommons.org/licenses/by/4.0/ This content is distributed under the terms of the Creative Commons Attribution 4.0 International license.

We next analyzed the differential gene expression pattern between the WT and *ktr3*Δ *cap6*Δ mutant upon TM treatment. We found that fewer genes showed changes in their expression levels in the *ktr3*Δ *cap6*Δ mutant than in the WT after TM treatment (Fig. 5B). In the WT strain, the expression of 423 genes (260 upregulated, 163 downregulated) was altered at least 2-fold in the presence of TM. In contrast, in the *ktr3*Δ *cap6*Δ mutant, 241 genes (193 upregulated, 48 downregulated) showed differential expression upon TM treatment (Fig. S6B). Those genes involved in the ER stress response mediated by the unfolded protein response (UPR) signaling pathway, such as genes involved in protein folding, glycosylation, and proteolysis (41), were induced upon TM treatment in both the WT and the *ktr3*Δ *cap6*Δ mutant but with slightly more upregulated genes in the *ktr3*Δ *cap6*Δ mutant (data not shown). The differential gene expression patterns were also reflected in the enrichment scores of DAVID analysis, additionally indicating that the small GTPase-mediated signal transduction category was upregulated in the WT upon TM treatment but not in the *ktr3*Δ *cap6*Δ mutant (Fig. S6C). Our transcriptome data also indicated that a set of genes in the calcineurin-Crz1 pathway (calcineurin-responsive zinc finger 1), which is coordinately regulated by phosphorylated Mpk1 in S. cerevisiae (45) and in C. neoformans (46), were upregulated by TM treatment in the C. neoformans WT strain but not in the *ktr3*Δ *cap6*Δ strain (data not shown). We further validated our transcriptome data by quantitative reverse transcription-PCR (qRT-PCR) analysis (Fig. S7A and B), strongly supporting that the blockage of *O*-glycan extension in the *ktr3*Δ *cap6*Δ strain results in defective Mpk1-mediated stress response signaling pathways.

10.1128/mbio.02112-22.9 FIG S7 Validation of differential gene expression observed in transcriptome data under the TM treatment condition. (A) qRT-PCR analysis of C. neoformans Mpk1-targeted gene expression. (B) qRT-PCR analysis of C. neoformans Crz1-targeted gene expression. (C) Analysis of sensitivity of C. neoformans *O*-glycan mutant strains to rapamycin. Download FIG S7, TIF file, 1.6 MB. Copyright © 2022 Thak et al. 2022 Thak et al. https://creativecommons.org/licenses/by/4.0/ This content is distributed under the terms of the Creative Commons Attribution 4.0 International license.

### Truncation of both major and minor *O*-glycans completely abolishes virulence in C. neoformans.

**FIG 6:**
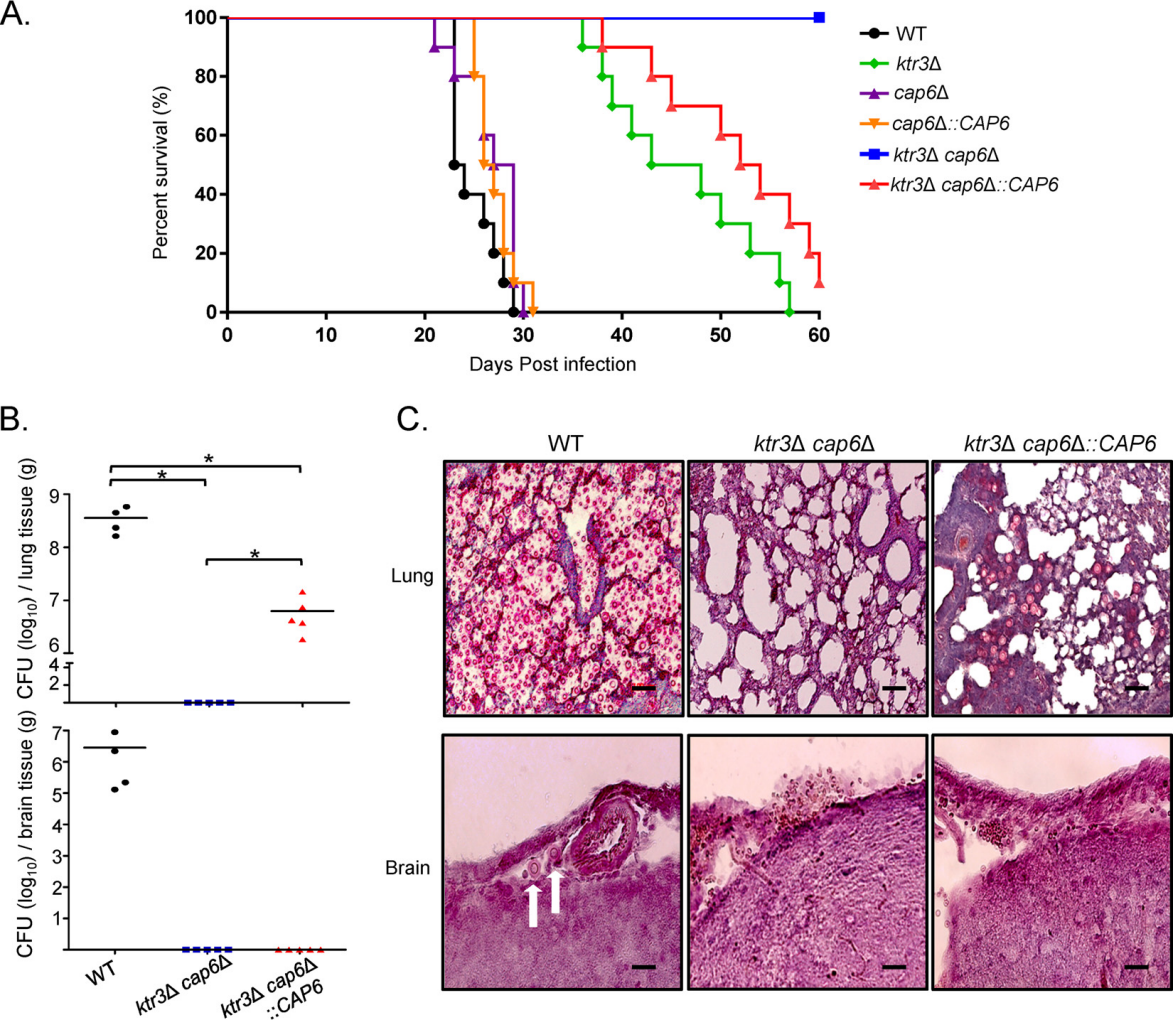
*In vivo* analysis of virulence of C. neoformans *O*-glycan mutants. (A) Survival analysis of mice infected C. neoformans. A/Jcr mice were infected with 10^5^ cells of WT, *ktr3*Δ, c*ap6*Δ, c*ap6*Δ::*CAP6*, *ktr3*Δ *cap6*Δ, and *ktr3*Δ *cap6*Δ::*CAP6* strains by intranasal instillation. Survival (%) was monitored for 8 weeks postinfection. (B) CFU of C. neoformans in the tissues of infected mice. The numbers of CFU per gram of lung and brain tissues were determined upon sacrifice of the mice infected with WT, *ktr3*Δ *cap6*Δ, and *ktr3*Δ *cap6*Δ::*CAP6* cells at day 21. *, *P* < 0.05 for WT versus *ktr3*Δ *cap6*Δ mutant, WT versus *ktr3*Δ *cap6*Δ::*CAP6* mutant, and *ktr3*Δ *cap6*Δ mutant versus *ktr3*Δ *cap6*Δ::*CAP6* mutant by one-way analysis of variance and Bonferroni’s *post hoc* tests. (C) Histopathological staining of infected lung and brain tissues with mucicarmine. White arrows indicate C. neoformans cells.

In our previous study, the truncation of the major *O*-glycans by the *KTR3* deletion was shown to severely attenuate virulence in animal models of C. neoformans infection (31). To assess the effect of an additional block of the extension of minor *O*-glycans on pathogenicity, we compared the virulence of *cap6*Δ, *ktr3*Δ, and *ktr3*Δ *cap6*Δ mutant strains to that of the WT in a murine inhalational model of systemic cryptococcosis. Although the single deletion of *CAP6* did not cause a detectable decrease in virulence of C. neoformans, the *ktr3*Δ *cap6*Δ mutant was more severely virulence attenuated than the *ktr3*Δ mutant. Even at 60 days postinfection (dpi), none of the mice infected with the *ktr3*Δ *cap6*Δ strain showed signs of clinical illness. Reintroduction of the WT *CAP6* gene into the *ktr3*Δ *cap6*Δ mutant restored virulence to the degree of the *ktr3*Δ mutant, indicating that the extended structures of both major and minor *O*-glycans contribute additively to the full pathogenicity of C. neoformans, although the role of minor *O*-glycans is not critical for virulence in the presence of major *O*-glycans (Fig. 6A).

Analysis of the lungs by quantitative culture at 21 dpi showed a marked decrease in fungal burden for the *ktr3*Δ *cap6*Δ strain compared to that of the WT and *ktr3*Δ *cap6*Δ::*CAP6* strains (Fig. 6B, top panel). Dissemination to the brain was also markedly reduced for both the *ktr3*Δ *cap6*Δ and *ktr3*Δ *cap6*Δ::*CAP6* strains, suggesting that *O*-glycans with extended structures are important for C. neoformans survival and proliferation in host environments after host infection. Histopathological analysis of mucicarmine-stained polysaccharide capsule of C. neoformans further supported the complete abolishment of the *ktr3*Δ *cap6*Δ cells in contrast to the extensive proliferation of the WT C. neoformans and a few surviving *ktr3*Δ *cap6*Δ::*CAP6* cells in the lung tissues (Fig. 6C, upper panel). In the brain tissues, a small number of C. neoformans cells was observed only in animals infected with the WT (Fig. 6C, lower panel).

### Extended *O*-glycans are required for host cell interaction in sequential steps of infection.

**FIG 7:**
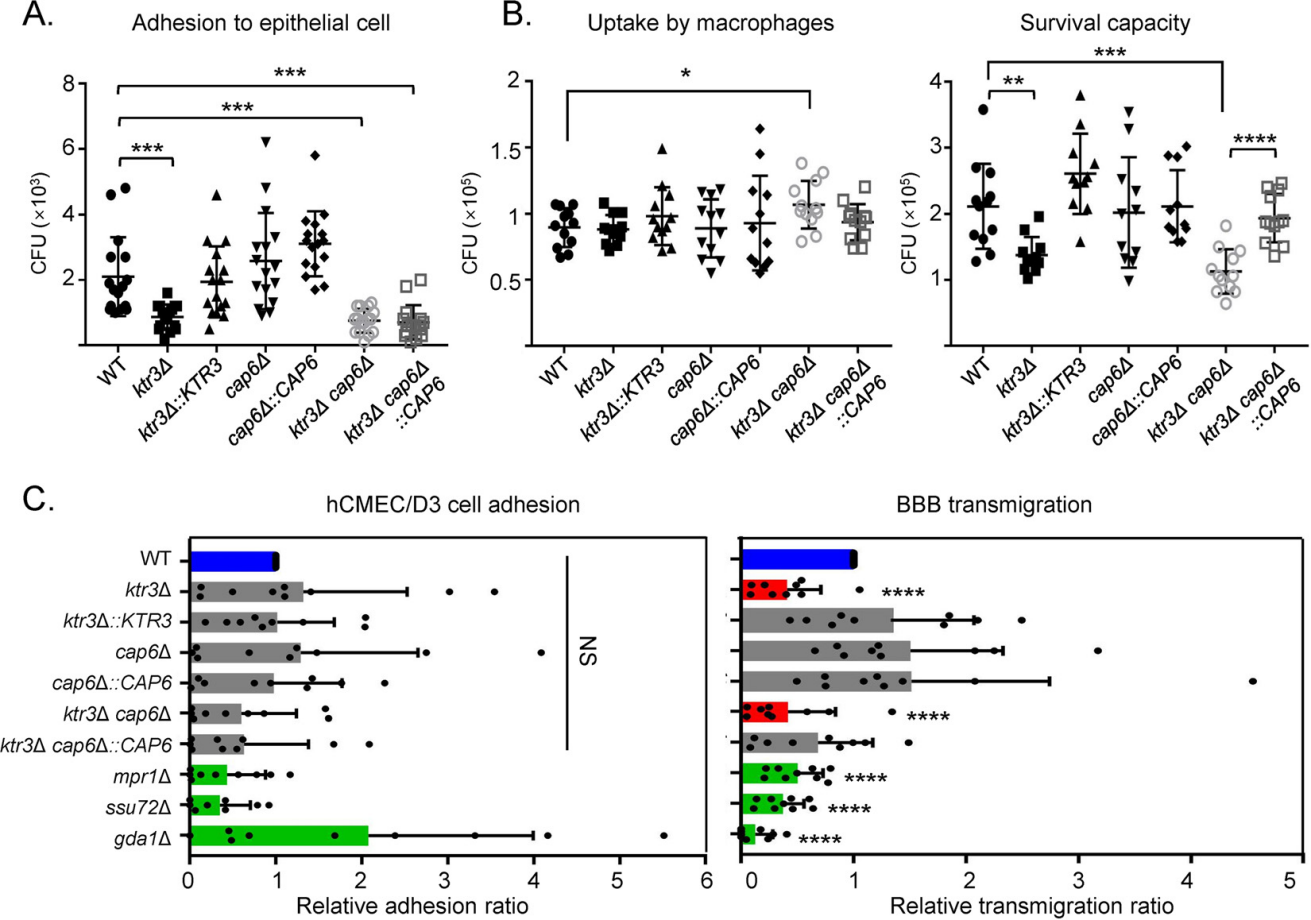
*In vitro* analysis of the interaction of C. neoformans *O*-glycan mutant strains with host cells. (A) Analysis of adherence to A549 (lung epithelial cells). The numbers of CFU of C. neoformans cells recovered from host cell lysates were counted after coincubation of C. neoformans cells with A549 lung epithelial cells at 37°C with 5% CO_2_ for 1 h. (B) Analysis of uptake by macrophages and survival capacity of C. neoformans in murine macrophage J774A.1. Opsonized C. neoformans cells were incubated with activated J774A.1 macrophages for 1 h. The numbers of CFU were counted to determine early phagocytosis. Survival capacity of C. neoformans by macrophages was determined by counting CFU in lysed macrophages. (C) Brain cell (hCMEC/D3) adhesion and BBB transmigration assays. Left panel, analysis of adherence to hCMEC/D3 cells. The numbers of CFU of C. neoformans cells recovered from host cell lysates were counted after coincubation of yeast cells with hCMEC/D3 at 37°C with 5% CO_2_ for 1 h. Right panel, analysis of transmigration assay. hCMEC/D3-coated plates were inoculated with yeast cells and incubated at 37°C in a CO_2_ incubator for 24 h, and the number of yeast cells passing through the hCMEC/D3-coated transwell was measured by CFU. C. neoformans *mpr1*Δ, *ssu72*Δ, and *gda1*Δ mutants were used as negative controls. *x* axes indicate the relative ratios of adhesion and transmigration, respectively. Each mutant was analyzed by three biologically independent experiments with three technical replicates. Data are presented as the mean value ± standard error of the mean (SEM). Statistical significance of differences between the WT (H99 strain) and each mutant (****, *P* < 0.0001) was calculated by a two-tailed Student's *t* test.

We defined the sequential infection steps involving *O*-glycan-mediated microbe-host interactions by analyzing the nature of the interaction of C. neoformans *O*-glycan mutant strains with lung epithelial cells, macrophage-like cells, and brain microvascular endothelial cells (Fig. 7). As the first step of host interaction during infection, we examined the efficiencies of adhesion of *O-*mannosylation mutant strains to A549 lung epithelial cells. The *ktr3*Δ, *ktr3*Δ *cap6*Δ, and *ktr3*Δ *cap6*Δ::*CAP6* strains showed significant reductions in epithelial cell adhesion efficiency, while the *cap6*Δ strain did not display apparent changes compared to the WT strain (Fig. 7A). For the subsequent infection step, we analyzed the uptake efficiency of C. neoformans mutant cells by macrophage-like cells (J774A.1) (Fig. 7B, left). The opsonic phagocytosis rates after 1 h of host-fungal cell coincubation were similar between WT and *O-*mannosylation mutant cells, indicating that the uptake efficiency by macrophages, the early step of phagocytosis, was not compromised by truncated *O*-glycans. To investigate the proliferation capability of the C. neoformans cells within the macrophage as a subsequent step of phagocytosis, we compared the intramacrophage survival capacities of WT and *O*-glycan mutant strains. The number of C. neoformans cells surviving within macrophages was remarkably reduced for the *ktr3*Δ *cap6*Δ mutant strain compared to the WT (~50%) (Fig. 7B, right). A more severely decreased survival capacity was observed in the *ktr3*Δ *cap6*Δ double mutant strain than the *ktr3*Δ single mutant, supporting the additive effect of minor *O*-mannosylation on C. neoformans intracellular survival.

Our recent study on genome-wide functional analysis of C. neoformans phosphatases indicated the involvement of the Golgi membrane-bound apyrases, Gda1 and Ynd1, in the interactions with the blood-brain barrier (BBB) (47). It was hypothesized that Gda1 and Ynd1 could modulate the mannosylation of both *N*-linked and *O*-linked glycoproteins as well as the addition of mannose residues to glycosphingolipids by affecting the antiport exchange ratio between GDP-mannose and GMP. To further investigate the requirement of *O*-linked glycoproteins for the interaction with the BBB, we compared the abilities of the *ktr3*Δ *cap6*Δ double mutant and WT cells to adhere to and cross cells associated with the BBB. We used an established *in vitro* transcytosis system, which consists of a transwell membrane coated with human cerebral microvascular endothelial cells (hCMEC/D3) separating a top compartment (blood side) and a bottom compartment (brain side) (48). Negative controls included C. neoformans strains with known defects in BBB penetration: (i) the *mpr1*Δ metalloprotease mutant (a brain infection-related factor that alters endothelium permeability [49]) and (ii) the *ssu72*Δ and *gda1*Δ phosphatase mutants (47). The *O*-glycan *ktr3*Δ and *ktr3*Δ *cap6*Δ mutant strains did not show any decreased ability of adhesion to the hCMEC/D3 cells. Notably, however, they showed a significantly reduced ability to traverse the BBB, suggesting that protein *O*-mannosylation is required for the full activity of BBB crossing but not adhesion to brain endothelial cells (Fig. 7C).

### Truncated *O*-glycans are defective in stimulating host immune response and in inducing host cell lysis.

**FIG 8:**
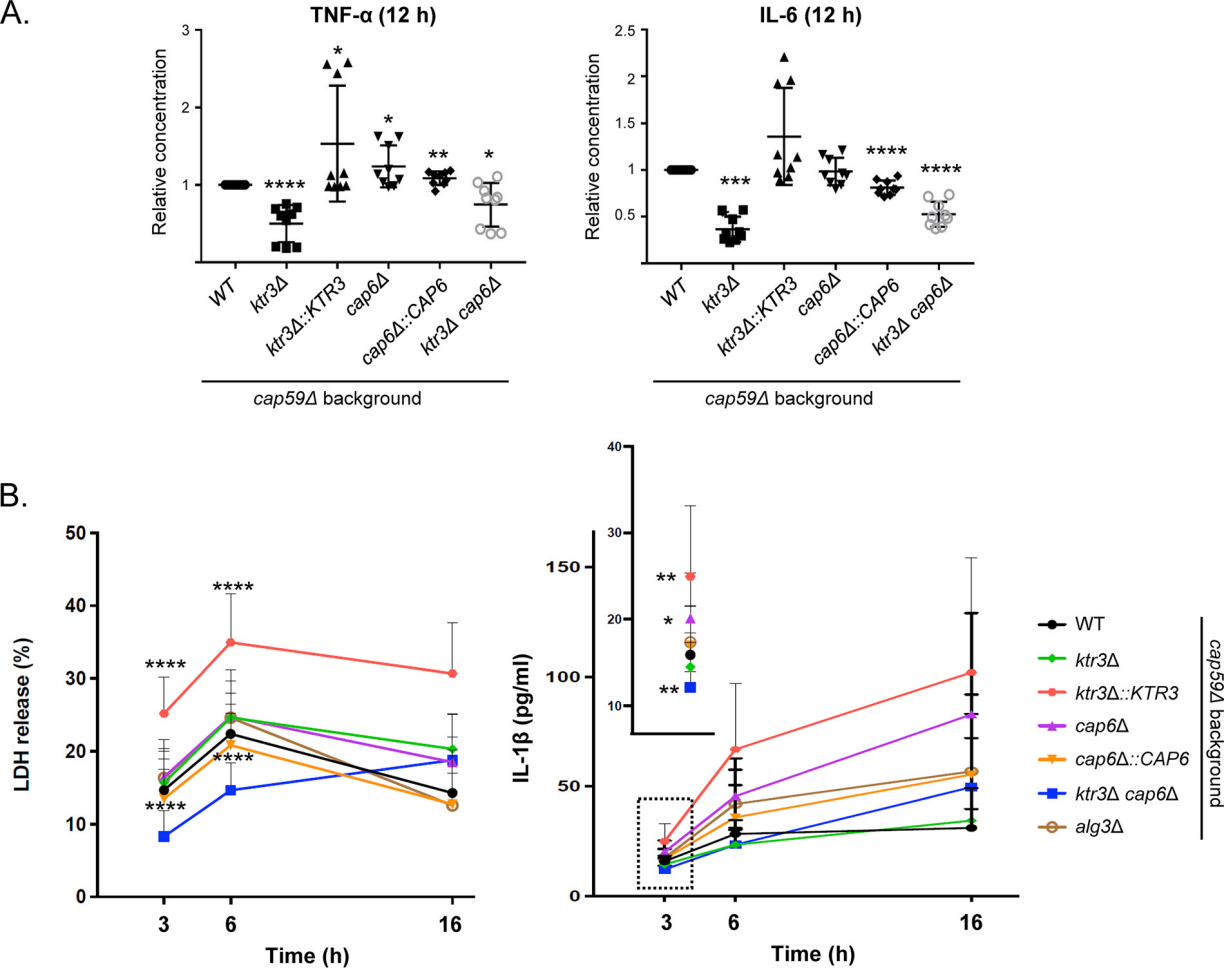
*In vitro* analysis of host immune response upon C. neoformans infection. (A) Analysis of cytokine secretion from host dendritic cells. C. neoformans cells were cocultivated with BMDCs for 12 h, and the levels of TNF-α and IL-6 secreted from dendritic cells were measured. Data represent means of triplicates from three independent experiments (*n* = 9). *y* axes indicate the relative concentrations of cytokines, which were calculated relative to those of the *cap59*Δ mutant. All the statistical analyses were performed using an unpaired two-tailed Student's *t* test (*, *P* < 0.05; **, *P* < 0.01; ***, *P* < 0.001; ****, *P* < 0.0001). (B and C) Analysis of host cell lysis and IL-1β release from BMDCs. BMDCs from WT mice were infected with cells of the WT, *O*-glycan mutants, and *alg3*Δ mutant in an acapsular background for the indicated times (3 h, 6 h, and 16 h). The cell culture supernatants were collected and assayed for LDH and IL-1β via ELISA (*, *P* < 0.05; **, *P* < 0.01; ***, *P* < 0.001; ****, *P* < 0.0001).

Dendritic cells are among the first immune cells that C. neoformans contacts in the infected lungs (50). To investigate whether the truncated *O*-glycans of the *ktr3*Δ *cap6*Δ mutant affect the response of host immune cells, we quantified the production of the proinflammatory cytokines tumor necrosis factor alpha (TNF-α) and interleukin 6 (IL-6) from bone marrow-derived dendritic cells (BMDCs) after C. neoformans infection. The BMDCs were coincubated with C. neoformans cells for 12 h, and the levels of cytokines from the cell culture supernatant were assayed (Fig. 8A). All strains were created in the *cap59*Δ background to minimize the antiphagocytic effect of the surface capsule, as performed previously (39). The BMDCs infected with either *ktr3*Δ or *ktr3*Δ *cap6*Δ mutant cells secreted lower levels of TNF-α and IL-6 during coincubation than those infected with the WT and *cap6*Δ cells. Interestingly, the *ktr3*Δ::*KTR3* complementation strain induced increased cytokine secretion compared with the WT, possibly due to the altered expression of reintroduced *KTR3*, which might generate *O*-glycan profiles that are not exactly the same as those of the WT (Fig. S2D).

Previous studies suggested that host cell pyroptosis, a cell escape strategy employed by fungal pathogens, is triggered by the *O*-mannosylated proteins assembled on the fungal cell surface (22–24). To examine whether defective *O*-mannosylation in C. neoformans also affects pyroptosis, we measured the levels of lactate dehydrogenase (LDH) and IL-1β released from BMDCs after 3 h, 6 h, and 16 h of coincubation with C. neoformans cells, including the WT, *O*-glycosylation mutants, and the *alg3*Δ *N*-glycosylation mutant strain (Fig. 8B). As previously reported for BMDCs infected with WT C. neoformans, LDH release showed almost the maximum value at 6 h without further increase while IL-1β release increased with prolonged incubation time (39). In contrast to the incubation with the *alg3*Δ mutant carrying truncated *N*-glycans, the BMDCs infected with the *ktr3*Δ *cap6*Δ mutant strain carrying truncated *O*-glycans showed reduced release of both LDH and IL-1β. However, the decreased release of IL-1β was evident only after 3 h of coincubation with the *ktr3*Δ *cap6*Δ strain, suggesting that *O*-glycans of C. neoformans are partly involved in triggering pyroptosis, which appears to occur early during infection (Fig. 8B). At later stages of coincubation (16 h), both LDH and IL-1β levels in the BMDCs infected with the *ktr3*Δ *cap6*Δ mutant were increased almost to the levels seen with the incubation with the WT C. neoformans cells (Fig. 8B), indicating that nonpyroptotic host cell lysis occurs at the late stage of infection without association with *O*-mannosylation.

**FIG 9:**
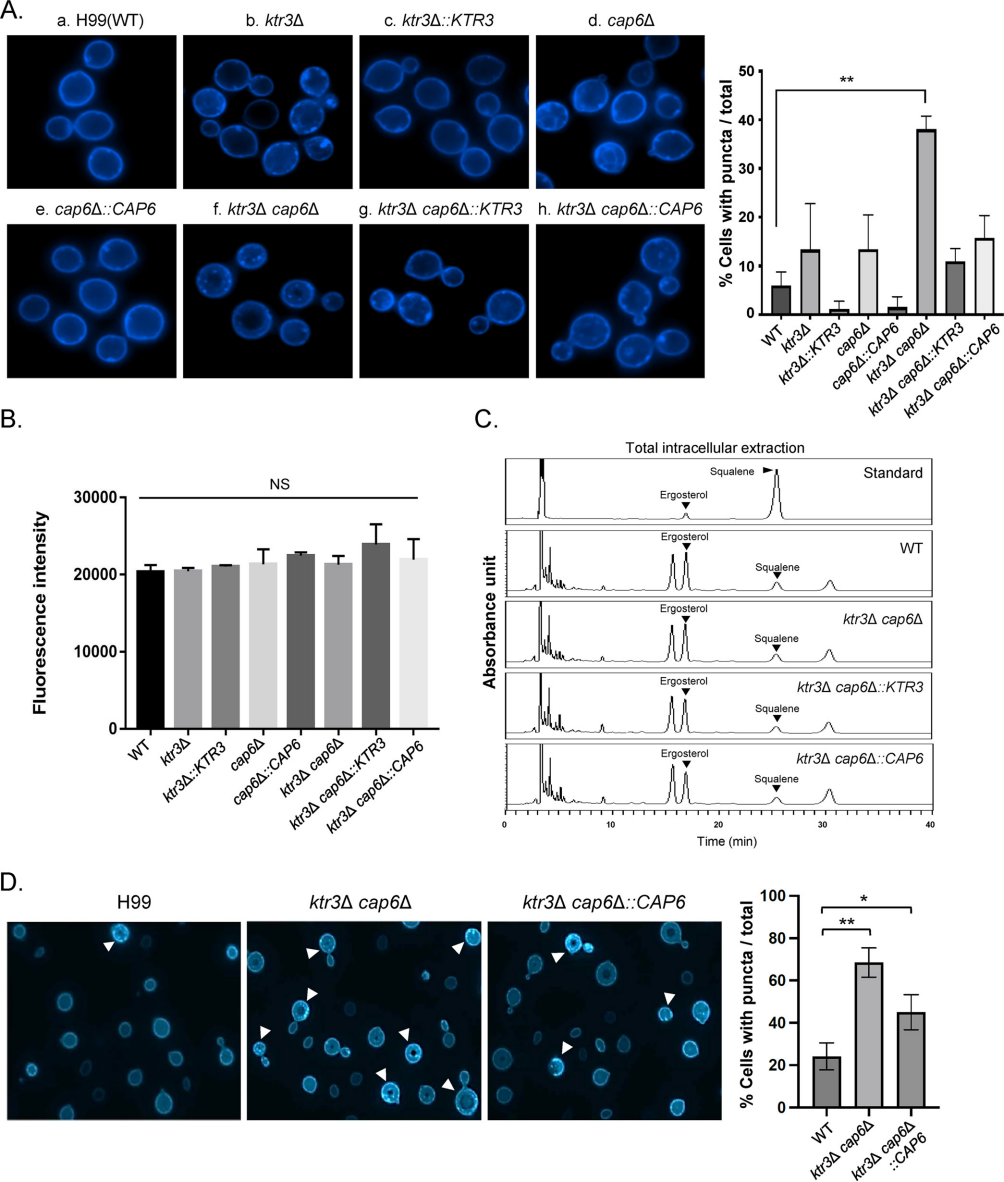
Localization and quantification of ergosterol in C. neoformans *O*-glycan mutant strains. (A) Filipin staining analysis of ergosterol localization. C. neoformans WT, *O*-mannosylation mutant, and complementation strains were incubated for 6 h at 30°C in YPD medium, fixed and stained with 10 μg/mL filipin, and monitored by a fluorescence microscope. Fluorescence images are shown of WT (a), *ktr3*Δ (b), *ktr3*Δ::*KTR3* (c), *cap6*Δ (d), *cap6*Δ::*CAP6* (e), *ktr3*Δ *cap6*Δ (f), *ktr3*Δ *cap6*Δ::*KTR3* (g), and *ktr3*Δ *cap6*Δ::*CAP6* (h) strains. The percentage of cells with puncta among the total cell population is shown. (B) Total fluorescence intensity of filipin staining. Fluorescence levels were quantitatively measured at a 485-nm wavelength using a fluorescence microplate reader (BioTek, Winooski, VT, USA). Data represent means of triplicates from two independent experiments. (C) HPLC profiles of the sterol samples from WT, *ktr3*Δ *cap6*Δ, *ktr3*Δ *cap6*Δ::*KTR3*, and *ktr3*Δ *cap6*Δ::*CAP6* strains. For the separation of ergosterol by HPLC, the proportion of solvent A (95% acetonitrile [ACN]) was maintained at 100% for 40 min. (D) Filipin staining analysis of C. neoformans WT, *ktr3*Δ *cap6*Δ, and *ktr3*Δ *cap6*Δ::*CAP6* strains cultivated for 18 h under host-mimicking tissue culture conditions, in which C. neoformans cells were incubated in CO_2_-independent tissue culture medium (TC medium; Gibco) with shaking at 37°C. The arrowheads indicate the C. neoformans cells showing increased cytoplasmic puncta.

Previous studies strongly indicated that *O*-mannosylation of cell wall mannoproteins plays a role in cell surface localization of ergosterol, which is important for the ability of S. cerevisiae and C. albicans to trigger pyroptosis (24, 51). To examine the effects of altered ergosterol localization in our *O*-mannosylation extension mutants, we stained the *ktr3*Δ, *cap6*Δ, and *ktr3*Δ *cap6*Δ mutants with filipin, a naturally fluorescent polyene antibiotic that binds to ergosterol, and analyzed them by fluorescence microscopy (Fig. 9A). Whereas the WT cells showed uniform staining of the plasma membrane (PM) (Fig. 9Aa), the *ktr3*Δ *cap6*Δ cells exhibited a pattern of scattered cytoplasmic and cell surface puncta (Fig. 9Af), resulting in significantly reduced cellular fluorescence signal at the PM. The *cap6*Δ and *ktr3*Δ single mutant strains showed an intermediate pattern of filipin staining between that of the WT and the *ktr3*Δ *cap6*Δ double mutant (Fig. 9Ab and d). The defects in ergosterol localization at the PM in the *ktr3*Δ *cap6*Δ strain were restored, accompanied by the disappearance of filipin-stained puncta, by complementation with either *CAP6* and *KTR3* (Fig. 9Ac, e, g, and h), as assessed by quantifying the percentage of cells with puncta.

We further investigated whether such altered ergosterol distribution might be associated with defective ergosterol biosynthesis by comparing the total fluorescence intensities of filipin staining (Fig. 9B) and the sterol profiles based on HPLC analysis (Fig. 9C). There was no apparent difference in total fluorescence intensity among WT and *O*-mannosylation mutant cells (Fig. 9B), indicating that total ergosterol levels were not detectably different between WT and *O*-mannosylation-detective strains. Considering that sterols are synthesized from the ER and transported, presumably by sterol transport proteins, to the sterol-enriched PM primarily via nonvesicular intracellular sterol pathways (52), we prepared sterol samples by employing the KOH-heptane extraction procedure, which is used frequently for measurement of total intracellular ergosterol production in fungal cells (53). When total intracellular sterol samples were analyzed by HPLC, the sterol profiles did not show notable difference between the WT, *ktr3*Δ *cap6*Δ, *ktr3*Δ *cap6*Δ::*KTR3*, and *ktr3*Δ *cap6*Δ::*CAP* strains (Fig. 9C). These nearly equivalent sterol profiles by HPLC analysis indicated that the *O*-mannosylation mutants do not have a defect in ergosterol biosynthesis. This suggests that the altered distribution of ergosterol observed by fluorescence microscopy likely results from defective trafficking of ergosterol. Abnormal ergosterol distribution was most notably observed in the *ktr3*Δ *cap6*Δ strain cultivated under host-mimicking conditions (TC medium, 37°C) (Fig. 9D).

## DISCUSSION

Protein *O*-mannosylation is a highly conserved process among eukaryotes by which PMTs in the ER catalyze the addition of mannose residues to Ser/Thr residues of their protein substrates (2). The further processing of *O*-mannosylation in the Golgi apparatus is differentially regulated in a species-specific manner, generating *O*-glycans with different structures and composition. In most yeasts studied thus far, the first mannose α-linked to Ser/Thr residues may be extended to form an α1,2-linked mannotriose (Manα1-2Manα1-2Manα1-Ser/Thr). This core structure is further processed differently according to the yeast species but mostly by the addition of mannose residues. The structures of the C. neoformans *O*-glycans are quite distinctive from those of other fungi, in that Manα1-2Manα1-6-Manα1-2Man mannotetraose is generated as major *O*-glycans by attaching the third mannose via α1,6-linkage instead of α1,2-linkage in C. neoformans, which is mediated by Hoc3, an α1,6-mannosyltransferase (31). Interestingly, C. neoformans also has oligomannoses containing xylose, Xyl_1_Man_2_ to Xyl_1_Man_4_ (X1M2 to X1M4), as minor *O*-linked glycans (Fig. 1B). We previously reported that the second α1,2-linked mannose residue in the major *O*-glycans is added to the first mannose by Ktr3 (31). Here, we demonstrated that Cap6, which was previously identified as α1,3-mannosyltransferase based on its homology to Cap59 involved in capsule biosynthesis, is responsible for the addition of the second mannose residue in α1,3-linkage, generating the minor *O*-glycans to be modified by addition of xylose. The subsequent addition of a third mannose in α1,6-linkage to minor *O*-glycans is mediated by Hoc1, another α1,6-mannosyltransferase, independently from major *O*-glycan biosynthesis in C. neoformans (31).

In S. cerevisiae, five membrane-spanning sensors, including Wsc1-3, Mid2, and Mtl2, detect external stress and trigger signal transduction cascades to maintain cell wall integrity. The structure of cell wall sensors has similar features, including a single transmembrane domain and cytoplasmic tails. The serine/threonine-rich (STR) region is especially highly *O*-glycosylated, and it is stretched by external stress and triggers activation of the CWI signaling pathway (40). In this study, we identified two novel cell wall stress sensors via *in silico* analysis in C. neoformans, Wml1 and Wml2 (see Fig. S3A in the supplemental material), which are modified independently by two different *O*-glycan synthesis pathways: Wml1 was mostly subject to Cap6-mediated *O*-mannosylation, while Wml2 was modified mainly by Ktr3-mediated *O*-mannosylation (Fig. 2B). Unlike with the fungal pathogens C. albicans and A. fumigatus, the C. neoformans cell wall sensors have not yet been reported, except that CNAG_03308 was previously predicted as a ScMtl1/ScMid2 homolog based on Pfam domain analysis (54). It is notable that C. neoformans Wml1 and Wml2 do not have the WSC1 domain or the MID2 domain, which are present in the cell wall sensors of other yeast species, including S. cerevisiae (Wsc1, Wsc2, Wsc3, Mid2, and Mtl2), C. albicans (Wsc1 and Wsc2), and Schizosaccharomyces pombe (Wsc1 and Mtl2) (Fig. S3B). Phylogenetic analysis revealed that the yeast Wsc and Mid proteins diverged from a common ancestor and evolved as a separate subfamily. It appears that C. neoformans Wml1 and Wml2 also evolved independently as a distinctive subfamily separated from other Wsc and Mid/Mtl homologs (Fig. S3C). C. neoformans Wml1 and Wml2 showed about 16% sequence identity to other cell wall stress sensors of several different yeasts (Fig. S3D), reflecting the difficult *in silico* identification based on sequency homology.

**FIG 10:**
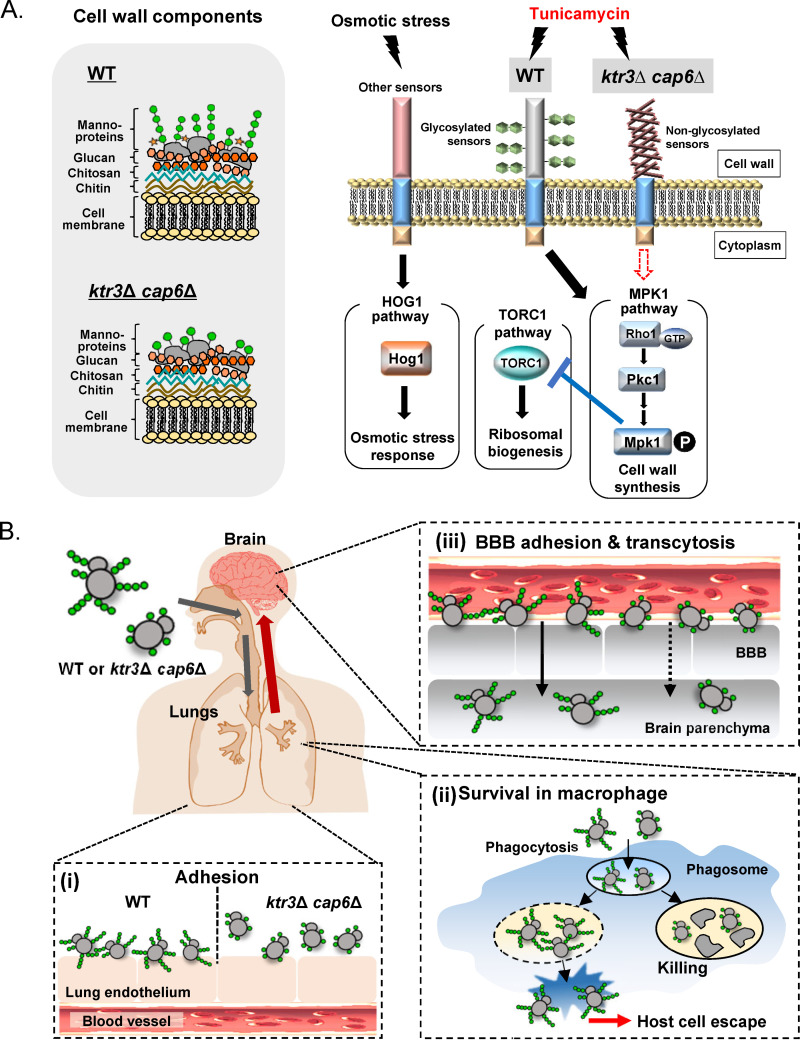
Schematic summary of the CWI pathway and interaction with host cells of C. neoformans WT and *ktr3*Δ *cap6*Δ mutant. (A) Schematic representation of the stress response signaling pathways mediated by Mpk1, Hog1, and TORC1. Extended *O*-mannosylation of cell surface sensors is required to induce Mpk1-mediated CWI signaling pathways and to repress TORC1 pathway but not for the induction of the HOG pathway. (B) Representative key steps in host cell-C. neoformans interactions. The extension of *O*-mannosylation in the Golgi apparatus generated is important for the interaction with host cells from early lung infection to late brain invasion, including (i) adherence to lung epithelial cells, (ii) survival in macrophage and host cell lysis, and (iii) transmigration to BBB.

We observed decreased phosphorylation of Mpk1 in the *wml1*Δ *wml2*Δ mutant strain upon TM treatment, suggesting the involvement of Wml1 and Wml2 as cell surface sensors in inducing CWI signaling in response to external stress (Fig. 4B). It is notable that the loss of Wml2 resulted in highly increased sensitivity to 1 M NaCl (Fig. S5B); in contrast, the *wml1*Δ, *wml2*Δ, and *wml1*Δ *wml2*Δ mutant strains exhibited no detectable growth defects under other stress conditions (Fig. S5A). Considering that Wml2 is *O*-mannosylated, which is required for its stability (Fig. 2B), the high salt sensitivity of *ktr3*Δ and *ktr3*Δ *cap6*Δ mutant strains might be partly attributed to the altered structures of *O*-glycans assembled on Wml2 (Fig. 2B). Hog1 phosphorylation and dephosphorylation patterns were not affected in the *ktr3*Δ *cap6*Δ mutant strain, indicating that the Wml2 serves as a sensor responding to salt stress independently from the HOG pathway (Fig. 10A). Further identification and functional characterization of C. neoformans cell surface sensors would provide in-depth insights into the C. neoformans signal processing in response to stresses, about which only limited information has been available on the cell surface sensors so far.

We observed that TM treatment evidently repressed expression of ribosomal protein genes in the WT, as previously reported in S. cerevisiae (55), but to a lesser extent than that in the *ktr3*Δ *cap6*Δ mutant (Fig. S6C). TORC1 (target of rapamycin complex 1), the central stress and growth controller, is involved in activation of ribosomal biogenesis (56). A recent study in S. cerevisiae reported that the TORC1 pathway is inhibited by TM exposure in cells of the WT strain but not in those lacking Slt2 (Mpk1), indicating that Mpk1 is required to activate the ER stress-protective mechanism through TORC1 inhibition (57). It was previously reported that Mtl1 *O*-mannosylation mediated by both Pmt1 and Pmt2 was important for cell survival under oxidative conditions and TOR blockade in S. cerevisiae (58). The C. neoformans *ktr3*Δ *cap6*Δ mutant also showed increased sensitivity to rapamycin (Fig. S7C). Together, the results suggest that the defective *O*-mannosylation in C. neoformans resulted in less efficient TORC1 inhibition upon TM treatment partly due to defective Mpk1 activation, which plays a role in inducing the ER stress-protective response by inhibiting TORC1 (Fig. 10A).

As major components of the fungal cell wall and PM, glycoproteins contribute to fungal pathogenicity and recognition by the host immunity (20). In our previous work on the *N*-glycosylation of C. neoformans, we reported that although the outer mannose chains of *N*-glycans are dispensable for the virulence of C. neoformans, an intact core *N*-glycan structure is required for C. neoformans pathogenicity (34, 39). The C. neoformans *ALG3*, *ALG9*, and *ALG12* deletion mutant strains, generating truncated core *N-*glycans, do not show defects in early stages of host cell interaction during infection, including attachment to lung epithelial cells, opsonic/nonopsonic phagocytosis, and phagosome maturation. However, the mutants defective in the core *N*-glycan biosynthesis exhibit decreased activity in inducing host cell death after phagocytosis, which is triggered as a mechanism of pulmonary escape and dissemination of C. neoformans, thus becoming inactive in causing fatal infection (39). Here, we presented a set of data indicating that the extended structure of *O*-glycans, generated by the Ktr3- or Cap6-mediated addition of the second mannose residue to the first mannose attached to target proteins in the Golgi apparatus, is important for proper interaction of C. neoformans with host cells at several steps during host infection (Fig. 10B). The *ktr3*Δ *cap6*Δ mutant showed decreased adhesion to lung epithelial cells, proliferation within macrophages, and reduced BBB crossing but normal efficiency of uptake by macrophages (Fig. 7). The *ktr3*Δ *cap6*Δ mutant also exhibited apparently reduced activity in inducing the immune response of primary host immune cells to secrete cytokines (Fig. 8). Particularly, the reduced release of both LDH and IL-1β from BMDCs upon infection with the *ktr3*Δ *cap6*Δ mutant indicates that cryptococcal *O*-glycans assembled on cell surface mannoproteins contribute to host cell pyroptosis, a cell escape strategy employed by fungal pathogens, although other factors are also required for full induction of host cell pyroptosis in C. neoformans. We observed the defective trafficking of ergosterol, which is considered an immunoreactive fungal molecule to trigger host cell pyroptosis (51), in the *ktr3*Δ *cap6*Δ cells (Fig. 9). Combined with the decreased resistance to stresses generated by host cell environments, the inefficient host cell escape of *ktr3*Δ *cap6*Δ cells could result in completely abolished virulence in C. neoformans (Fig. 10).

Besides decreased ergosterol localization in the plasma membrane of the *ktr3*Δ *cap6*Δ mutant strain, we examined the possibility that the defective *O*-glycan structure might induce altered physiochemical properties of the cell surface by changing other cell wall components (Fig. S8). Although no apparent difference in the total amount and overall lengths of *N*-glycans was observed between the WT and *ktr3*Δ *cap6*Δ mutant, the *N*-glycan profile of the *ktr3*Δ *cap6*Δ mutant showed decreased neutral glycan peaks (*P = *0.0558) with increased acidic glycan peaks (*P = *0.0553) compared to that of the WT strain (Fig. S8A and S8B). We observed a slightly increased amount of chitin (Fig. S8C) in the *ktr3*Δ *cap6*Δ mutant, which might reflect the compensation mechanism of defective cell wall integrity. Thus, the defective host interaction observed in the *ktr3*Δ *cap6*Δ mutant might result mainly from the combined effect of the truncated structure of *O*-glycans, which might be directly recognized with host glycan-binding proteins and the defective localization of ergosterol, which is required for triggering host immune response. Further studies to elucidate the underlying mechanisms on how the specific structures of C. neoformans *O*-glycans are recognized by host cells and how *O*-glycans are involved in ergosterol trafficking would facilitate our understanding of the glycan-based modulation of host-pathogen interactions in infectious fungi.

10.1128/mbio.02112-22.10 FIG S8 Analysis of *N*-linked glycan profiles and cell wall components in the *O*-glycan mutant strains. (A) HPLC profiles of acidic and neutral *N*-glycans of the WT and *O*-glycan mutant strains. For the separation of acidic and neutral *N*-glycans, the proportion of solvent B was maintained at 10% for 5 min and then increased linearly to 90% over 60 min. (B) Percent areas of acidic and neutral *N*-glycans of the WT and *O*-glycan mutant strains. (C) Flow cytometry analysis of cell wall components. WT and mutant strains were incubated for 16 to 18 h at 30°C in YPD medium, fixed, labeled with CFW, and analyzed by flow cytometry. Unstained cells were sorted as controls to determine positive labeling. Download FIG S8, TIF file, 1.4 MB. Copyright © 2022 Thak et al. 2022 Thak et al. https://creativecommons.org/licenses/by/4.0/ This content is distributed under the terms of the Creative Commons Attribution 4.0 International license.

This study demonstrated the critical roles of *O*-glycan structures in the pathogenicity of C. neoformans. Even though PMT family inhibition is a possible strategy to block the initiation of *O*-glycan assembly on proteins, the PMT family is highly conserved through eukaryotes from yeast to Homo sapiens, and there is no single Pmt inhibitor with activity against all the PMT family enzymes on their diverse acceptor protein substrates. In contrast, there is no human homolog for the fungal mannosyltransferases working in the Golgi apparatus, such as C. neoformans Cap6 and Ktr3. Furthermore, the addition of the second mannose residue by Ktr3 and Cap6 in the Golgi apparatus occurs commonly in all the acceptor proteins subjected to *O*-mannosylation. Thus, the fungus-specific α1,3-/α1,2-mannosyl transferases involved in *O*-mannosylation might be very promising targets for the development of new antifungal agents.

## MATERIALS AND METHODS

### Strains, media, plasmids, and primers.

The C. neoformans strains constructed and used in this study are listed in Table S1A in the supplemental material. Yeast cells were generally cultured in YPD medium (1% [wt/vol] yeast extract, 2% [wt/vol] peptone, 2% [wt/vol] dextrose) with shaking (220 rpm) at 30°C. The plasmids and primers used in this study are listed in Tables S1B and C, respectively. C. neoformans transformants were selected on YPD solid medium containing 100 μg/mL nourseothricin acetyltransferase (Jena Bioscience), referred to here as YPD_NAT_, or on YPD solid medium containing 200 μg/mL G418 disulfate (Duchefa), referred to here as YPD_G418_, or on YPD solid medium containing 100 μg/mL hygromycin B (Sigma), referred to here as YPD_HYB_. Gene sequence information was obtained from the C. neoformans serotype A genome database (NCBI).

10.1128/mbio.02112-22.2 TABLE S1 (A) Strains used in this study. (B) Plasmids used in this study. (C) Oligonucleotides used in this study. Download Table S1, DOCX file, 0.05 MB. Copyright © 2022 Thak et al. 2022 Thak et al. https://creativecommons.org/licenses/by/4.0/ This content is distributed under the terms of the Creative Commons Attribution 4.0 International license.

### *O*-Glycan analysis by HPLC.

For release of the *O*-glycans, the cell wall mannoproteins (cwMPs) were prepared as described in previous studies (31, 59). Briefly, the completely dried cwMPs (50 μg) were resuspended in 100 μL of hydrazine monohydrate (Tokyo Chemical Industry), and the mixture was incubated at 60°C for 4 to 6 h. The reactants were dried to remove the hydrazine monohydrate, and the pellets were dissolved in 100 μL of saturated NaHCO_3_ (Sigma), mixed with 10 μL of (CH_3_CO)_2_O, and incubated on ice for 30 min without shaking. The *O*-glycans were purified by using Dowex 50WX8-400 resins (H^+^ form; Sigma), and the isolated *O*-glycans were labeled with 2-aminobenzoic acid (2-AA; Sigma) and purified using a cyano base cartridge (Bond Elut-CN-E; Agilent) (100 mg). The HPLC analysis of the 2-AA-labeled *O*-glycan was conducted on a TSKgel Amide-80 column (0.46 by 25 cm, 5 μm; Tosoh, Tokyo, Japan) at a flow rate of 1.0 mL/min. 2-AA-Oligosaccharides were detected with a 2475 fluorescence detector (Waters) at excitation and emission wavelengths of 360 and 425 nm, respectively. Data were collected using Empower 2 chromatography data software (Waters).

### *In silico* bioinformatics analysis to identify cell surface sensor proteins from the C. neoformans proteome.

Two candidates of cell wall stress sensors in C. neoformans and various cell wall proteins related to the CWI pathway in S. cerevisiae, C. albicans, and S. pombe were analyzed using InterPro (https://www.ebi.ac.uk/interpro/), SignalP (https://services.healthtech.dtu.dk/service.php?SignalP-5.0), TMHMM (https://services.healthtech.dtu.dk/service.php?TMHMM-2.0), YinOYang (https://services.healthtech.dtu.dk/service.php?YinOYang-1.2) and NetOGlyc 4.0 (https://services.healthtech.dtu.dk/service.php?NetOGlyc-4.0), as follows. First, C. neoformans proteins containing a region of 40 amino acids in which the percentage of Ser/Thr was ≥40% were identified from the C. neoformans proteome, resulting in 958 proteins (12% of the C. neoformans proteome). Then, a more restrictive search was carried out for membrane-attached proteins containing a single-pass transmembrane domain in the C-terminal region, narrowing to 80 proteins from the C. neoformans database (1% of the C. neoformans proteome). The third and fourth search criteria were the absence of a GPI-anchor domain but the presence of a signal peptide at the N-terminal region, respectively. This iterative screen resulted in 12 candidate proteins. Additional screening of these 12 proteins was performed with the NetOGlyc server (https://services.healthtech.dtu.dk/service.php?NetOGlyc-4.0) to predict *O*-glycosylation sites, and two putative cell wall sensors of C. neoformans, CNAG_01255 and CNAG_03328, were finally selected. Multiple sequence alignments and percentage identity were analyzed by the CLUSTALW method of the DNASTAR MegAlign program. A phylogenetic tree based on the sequences of the cell wall stress sensor homologs was constructed using the maximum likelihood tree of the MEGA X software.

### Animal study.

Animal studies were conducted at the Chung-Ang University Animal Experiment Center and were approved by the Ministry of Food and Drug Safety (MFDS; South Korea). The experimenter completed an animal experimental education and followed the guidelines. C. neoformans strains (H99, *ktr3*Δ, *cap6*Δ, *ktr3*Δ *cap6*Δ, and *ktr3*Δ *cap6*Δ::*CAP6* strains) were washed with sterile phosphate-buffered saline (PBS) and then resuspended in sterile PBS at a density of 2 × 10^6^ cells per mL. Ten mice (6-week-old female A/J Slc mice, 16 to 18 g; Japan SLC) per strain were infected via intranasal instillation of 10^5^ cells (in 50 μL). Following infection, the mice were weighed and monitored once daily and then sacrificed using CO_2_ when they lost 30% of the original body weight rapidly. Kaplan-Meier survival curves were generated by using Prism 5.02 (GraphPad Software).

For the fungal burden assay, five mice per strain were infected with C. neoformans strains (H99, *ktr3*Δ *cap6*Δ, and *ktr3*Δ *cap6*Δ::*CAP6* strains). The lungs and brains of C. neoformans-infected mice (*n* = 5 for each strain, except *n* = 4 for WT strain due to death on day 20) were dissected on day 21. Lungs and brains were excised, and half organ portions were analyzed by quantitative culture of CFU. For histopathological analysis, half lung and brain samples were fixed and mucicarmine staining was performed. After slide preparation, each sample was observed using a Zeiss Axioscope (A1) equipped with an AxioCam MRm digital camera.

### *In vitro* host cell interaction analysis.

To analyze adhesion efficiency of C. neoformans cells to epithelial lung cells (A549), A549 cells (2 × 10^5^) were seeded into 24-well plates in Dulbecco’s modified Eagle’s medium (DMEM; Gibco) supplemented with 10% fetal bovine serum (FBS) and cultivated at 37°C in 5% CO_2_ for 18 h. Yeast cells (2 × 10^6^) were added to each well (C. neoformans/A549 ratio, 10:1) and coincubated for 1 h. Then, nonadherent yeast cells were removed by washing with PBS, and the A549 cells were lysed in distilled water. The number of yeast cells was assessed by CFU analysis in three independent experiments. The uptake of C. neoformans cells by macrophages was analyzed with the C. neoformans cells opsonized with 10 μg/mL mouse immunoglobulin G 18B7 antibody (kindly gifted by Arturo Casadevall, Johns Hopkins School of Public Health) at 37°C for 1 h. The macrophage-like J774A.1 cells (1 × 10^5^) were seeded into 96-well plates in DMEM supplemented with 10% FBS and cultivated at 37°C in 5% CO_2_ for 18 h. Opsonized C. neoformans cells (1 × 10^5^) were added to activated macrophages (C. neoformans/J774A.1 ratio, 1:1) and incubated with serum-free DMEM. After 1 h of coincubation, nonphagocytized yeast cells were removed by washing and the macrophages were lysed in distilled water. The number of C. neoformans cells phagocytized by macrophages was assessed by CFU analysis in three independent experiments. To analyze the fungal cell survival in macrophages (J774A.1), after 1 h of coincubation, each well was washed to remove extracellular yeast cells. Then, 100 μL of macrophage medium was added to each well and the C. neoformans-infected macrophages were incubated for 24 h. Then, macrophages were lysed in distilled water by vigorous pipetting, and the fungal cells were collected, serially diluted, and plated onto a YPD plate to assess the number of viable C. neoformans cells.

### Analysis of host immune response by cytokine and LDH analysis.

For analysis of secreted cytokines, activated BMDCs were prepared as previously described (39) with slight modification. The BMDCs (1 × 10^5^ cells/well) were inoculated into 96-well plates (in RPMI medium plus 10% heat-inactivated FBS) and incubated at 37°C in 5% CO_2_ for 1 h. The BMDCs were then incubated with cryptococcal cells (1 × 10^6^ cells/well) for 12 h, and the supernatants were collected and centrifuged at 17,000 × g for 5 min. Released levels of TNF-α and IL-6 were analyzed by enzyme-linked immunosorbent assay using mouse TNF-α ELISA Max set deluxe kits and mouse IL-6 ELISA Max set deluxe kits (BioLegend), according to the manufacturer’s instructions.

For analysis of host cell lysis, C. neoformans cells were washed twice with PBS, counted, and added to BMDCs at a concentration of 2 × 10^6^ cells/well. After incubation for the indicated time (3 h, 6 h, or 16 h), supernatants were collected and centrifuged at 17,000 × *g* for 5 min. Levels of released IL-1β were analyzed by enzyme-linked immunosorbent assay (ELISA) using mouse IL-1β ELISA Max set deluxe kits (BioLegend), according to the manufacturer’s instructions. Levels of LDH enzyme released into the cell culture supernatant were measured as a colorimetric change using an LDH cytotoxicity detection kit (Pierce) according to the manufacturer’s protocol. The plate was read at 490 nm using a microplate reader after 30 min of incubation at room temperature. The absorbance was measured, and percent cytotoxicity was calculated for the samples.

### hCMEC/D3 cell adhesion and BBB transmigration assays.

*In vitro* BBB adhesion and transmigration assays were performed as previously described (60). Briefly, a human brain microvascular endothelial cell (HBMEC) line (hCMEC/D3 cell line; Merck) was cultured at 37°C with 5 % CO_2_ in EGM-2 medium (Lonza). For the BBB adhesion assay, 5 × 10^4^ hCMEC/D3 cells in EGM-2 medium were seeded on collagen-coated 12-well plates (BD Falcon) and cultured until the monolayer became confluent. A day before C. neoformans inoculation, the EGM-2 medium (2.5% human serum) was replaced with EGM-2 medium (1.25% human serum), and integrity of tight junction formation was measured as transendothelial electrical resistance (TEER) using an epithelial volt per ohm meter (EVOM^2^ device; World Precision Instruments). hCMEC/D3 cells were coincubated with 5 × 10^5^ fungal cells for 24 h, and then the endothelial barrier was washed to remove nonadherent cells and lysed with water to determine the number of cells that adhere to the barrier.

For the BBB crossing assay, hCMEC/D3 cells were seeded in a transwell chamber and cultured as described as above. C. neoformans cells were inoculated onto the top of the porous membranes. After 24 h of incubation, the medium in the bottom chambers was collected and the number of yeast cells passing through the porous membrane was measured by counting the CFU. The BBB adhesion or migration ratio was calculated by dividing the output CFU of each tested strain by that of the WT.

### Ergosterol staining analysis.

For ergosterol staining, C. neoformans cells at an OD_600_ of 0.3 were cultivated in 25 mL YPD at 30°C for 6 h. The cell pellets were washed with PBS and fixed with 4% paraformaldehyde (PFA) solution for 20 min on an 18-rpm rotator. After washing with PBS, the cells were stained with 10 μg/mL filipin III (Cayman Chemical, Ann Arbor, MI) in the dark for 5 min on an 18-rpm rotator. After washing with PBS, cell pellets were resuspended in 500 μL PBS and observed immediately. Cells were visualized with an Eclipse Ti-E fluorescence microscope (Nikon) equipped with a Nikon DS-Qi2 camera and a Plan Apo VC 100× oil differential interference contrast (DIC) N2 (numerical aperture, 1.4) lens using an excitation of 360 nm and an emission of 480 nm. Images were processed based on the NIS-Elements microscope imaging software (Nikon).

### Ergosterol extraction and analysis.

Total intracellular sterols were extracted using KOH-heptane (53). Briefly, C. neoformans cells, cultivated in YPD liquid medium at 30°C for 48 h, were washed three times with PBS. Subsequently, cell cultures were centrifuged and resuspended in 1 mL KOH-H_2_O-ethanol (EtOH) per 0.1 g wet weight of cell pellet. After incubation 1 h at 85°C, 500 μL heptane was added to the mixture and stirred using a Precellys 24 tissue homogenizer (Bertin Technologies) at 6,500 rpm for 30 s. The mixtures were centrifuged at 13,000 rpm for 15 min, and the supernatants were collected and freeze-dried overnight.

For ergosterol analysis, the total intracellular sterol extract was dissolved in 250 μL of acetone. After filtration, the samples were subjected to HPLC analysis using a COSMOSIL 5C18-PAQ packed column (120 Å, 5 μm, 4.6 mm inner diameter by 250 mm) at a flow rate of 1.0 mL/min. Ergosterol was detected with a Waters 2487 absorbance detector at wavelengths of 203 nm. Data were collected using Empower 2 chromatography data software (Waters).

### Data availability.

Raw RNA sequencing data have been submitted to the NCBI GEO database under accession no. GSE198875.
