# Supplementary figures and images for: Extension of O-Linked Mannosylation in the Golgi Apparatus Is Critical for Cell Wall Integrity Signaling and Interaction with Host Cells in Cryptococcus neoformans Pathogenesis
Source: mBio. 2022 Nov 21;13(6):e02112-22. doi: 10.1128/mbio.02112-22 (PMC9765558; doi:10.1128/mbio.02112-22)

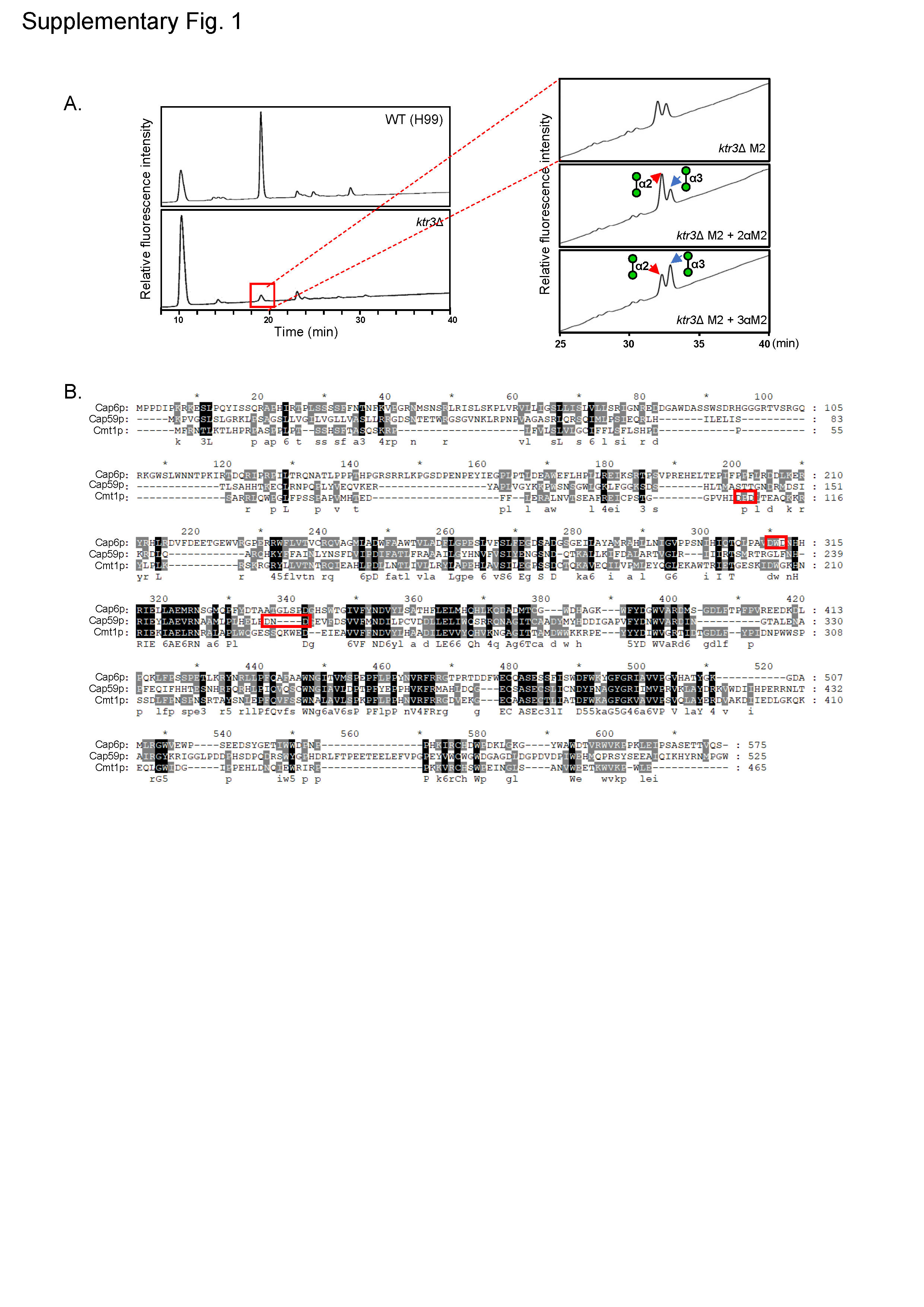

Supplement: FIG S1 [file mbio.02112-22-s0001.tif]

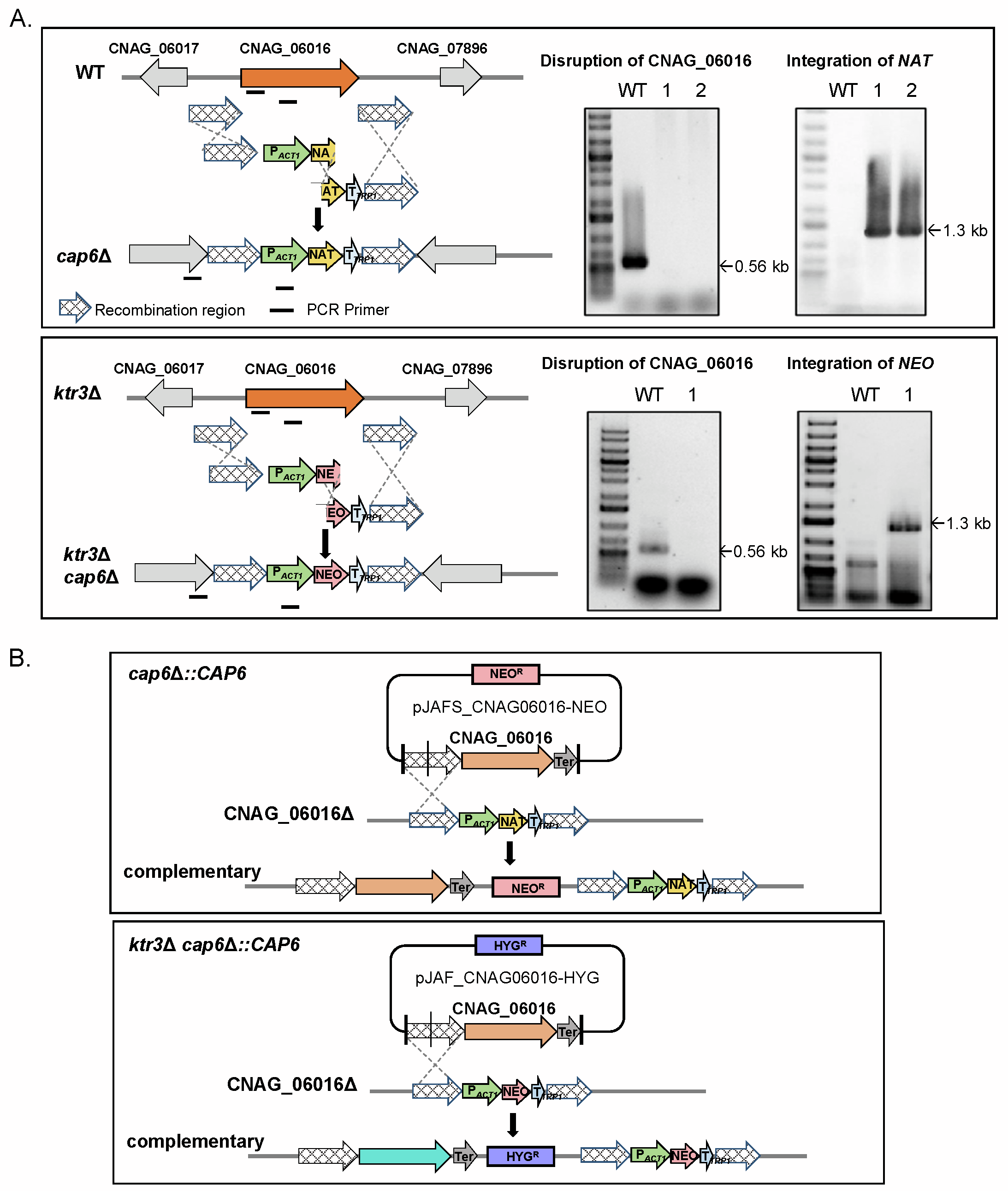

Supplement: FIG S2 [file mbio.02112-22-s0002.tif]

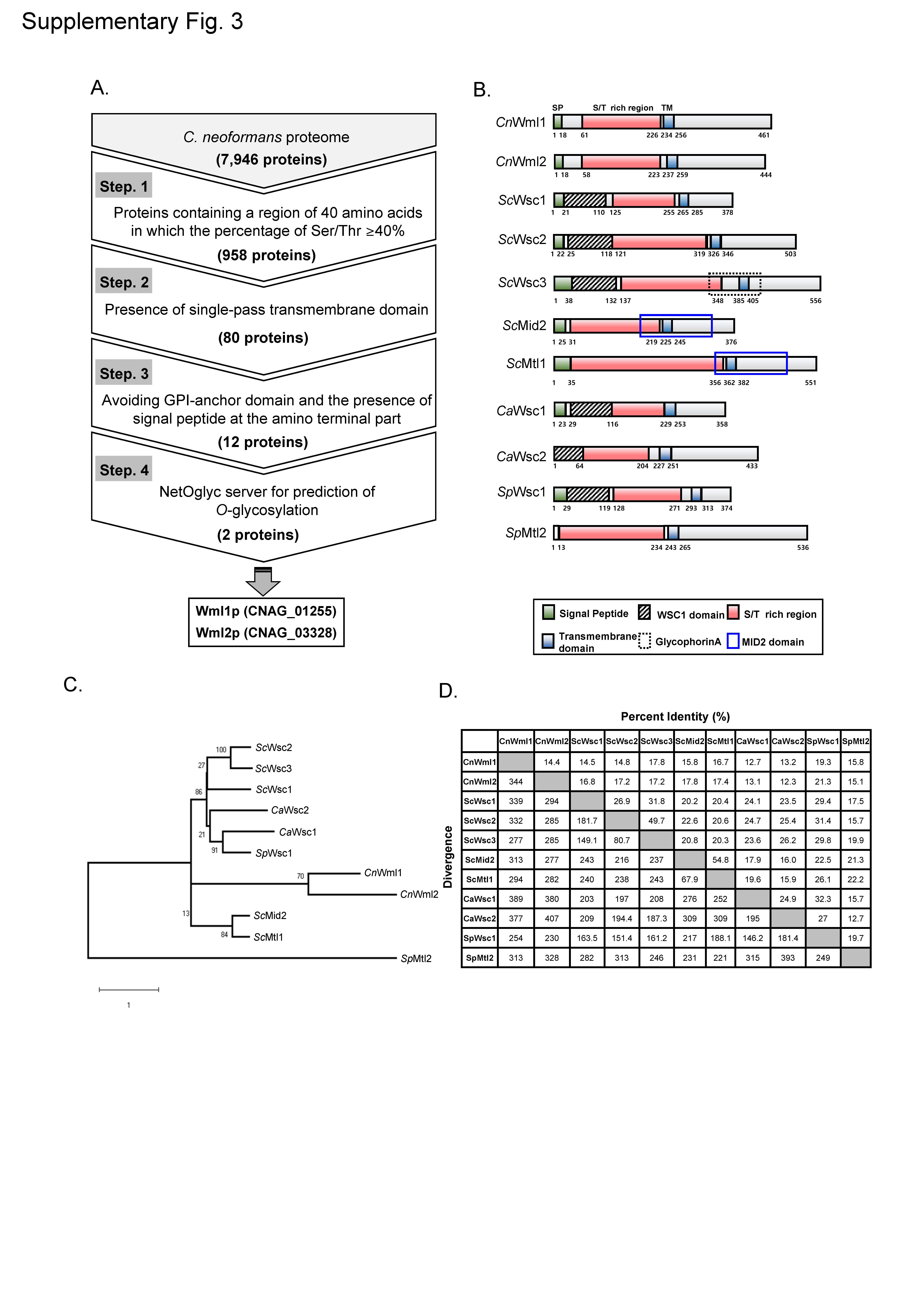

Supplement: FIG S3 [file mbio.02112-22-s0003.tif]

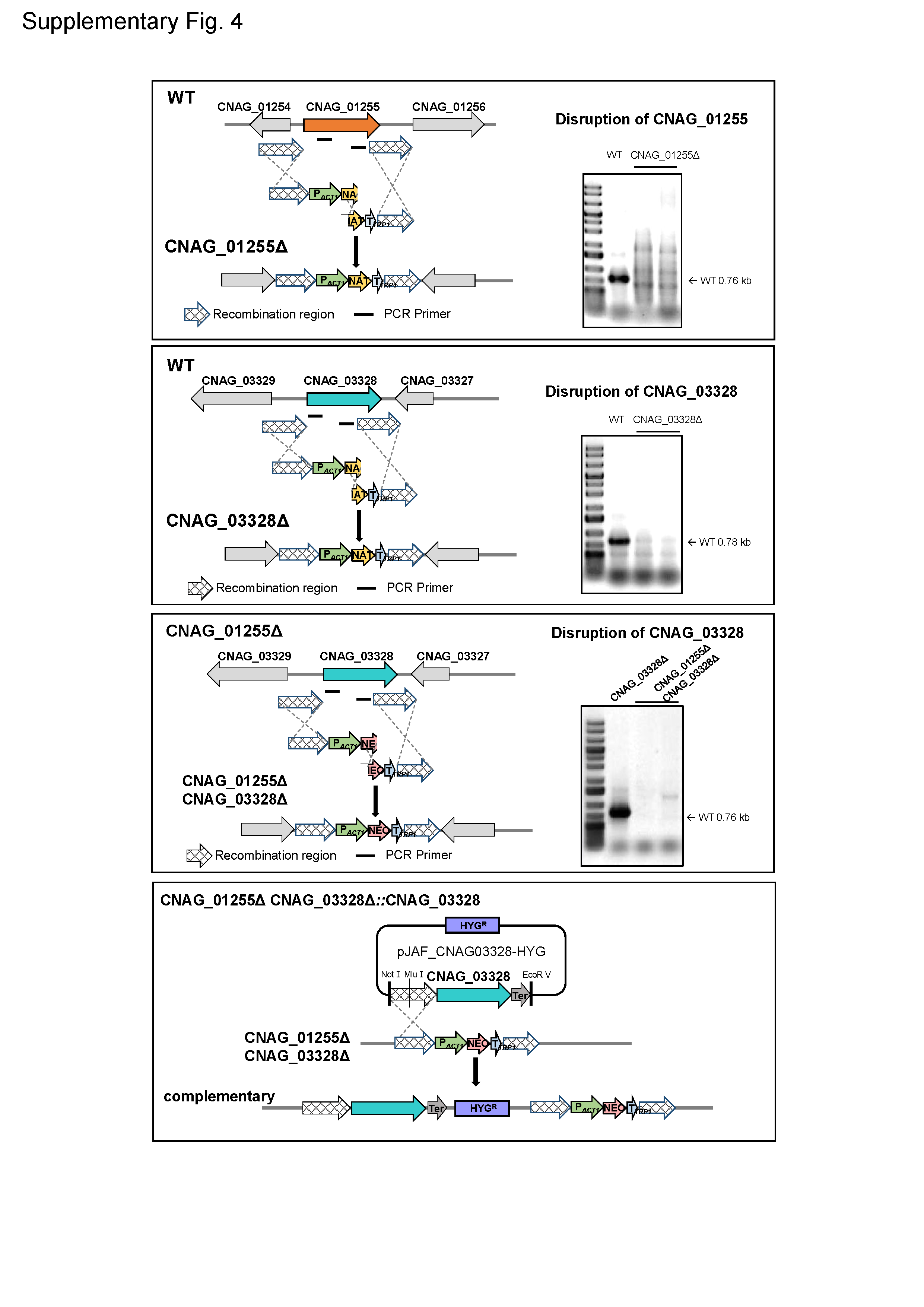

Supplement: FIG S4 [file mbio.02112-22-s0004.tif]

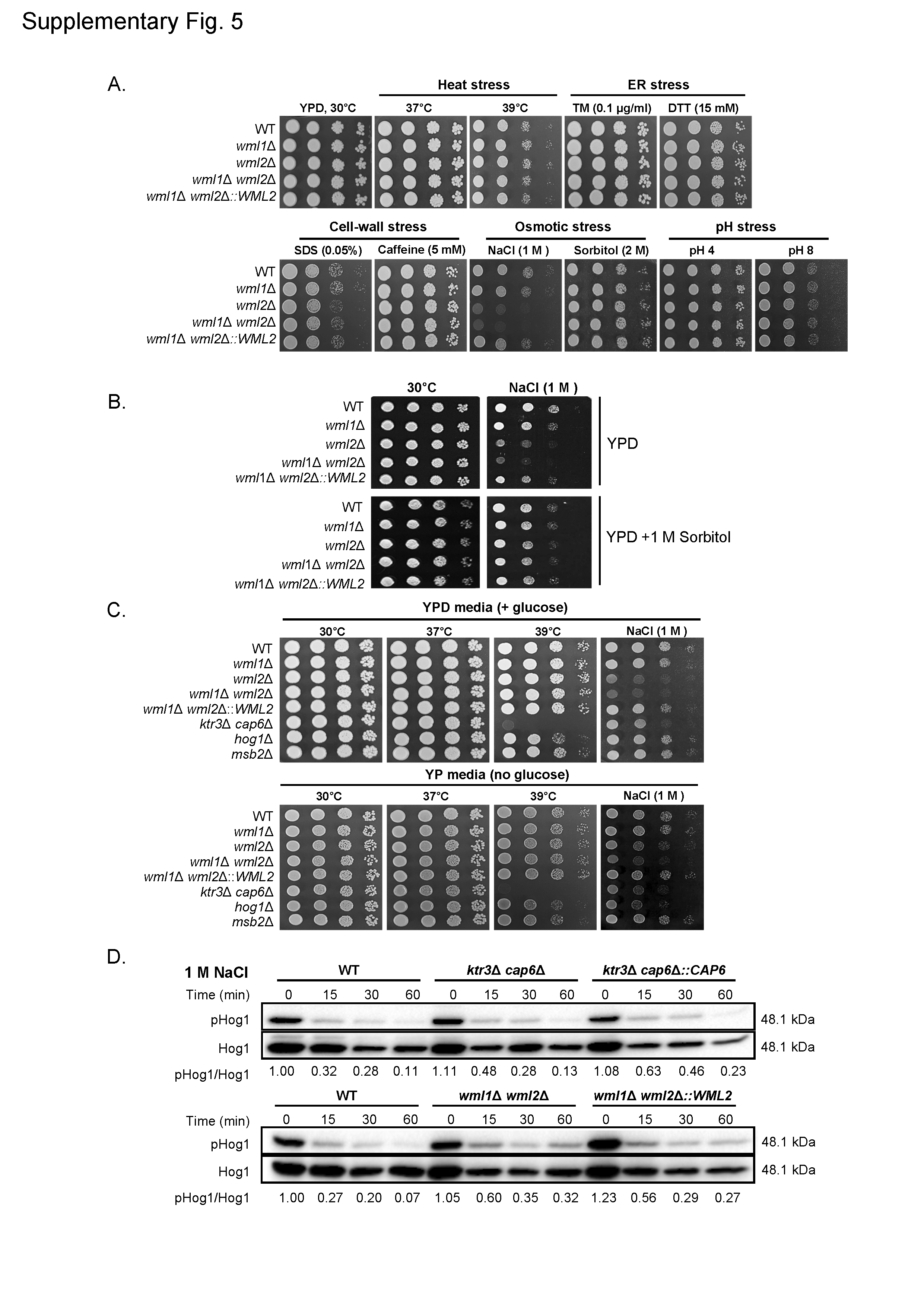

Supplement: FIG S5 [file mbio.02112-22-s0005.tif]

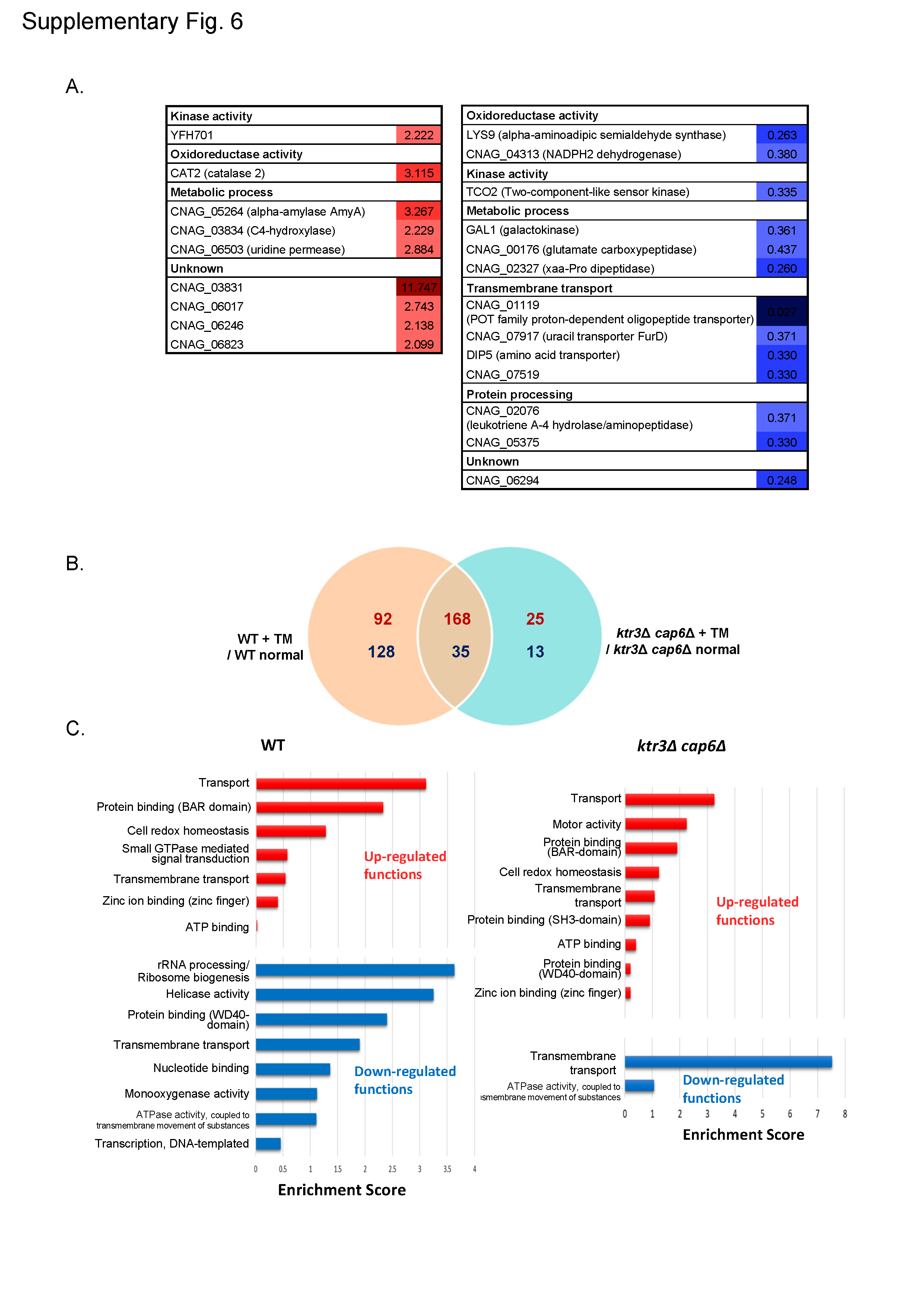

Supplement: FIG S6 [file mbio.02112-22-s0006.tif]

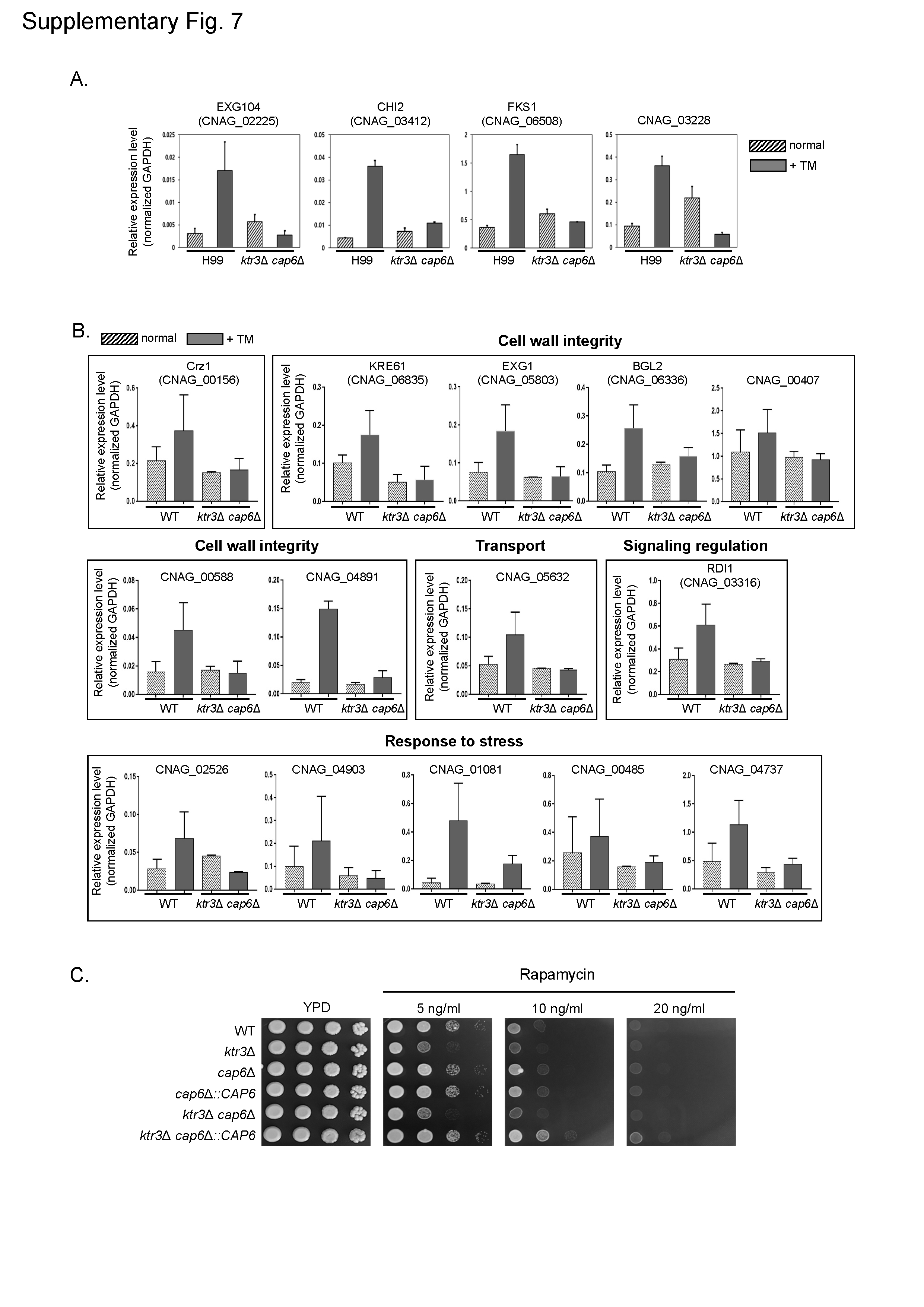

Supplement: FIG S7 [file mbio.02112-22-s0007.tif]

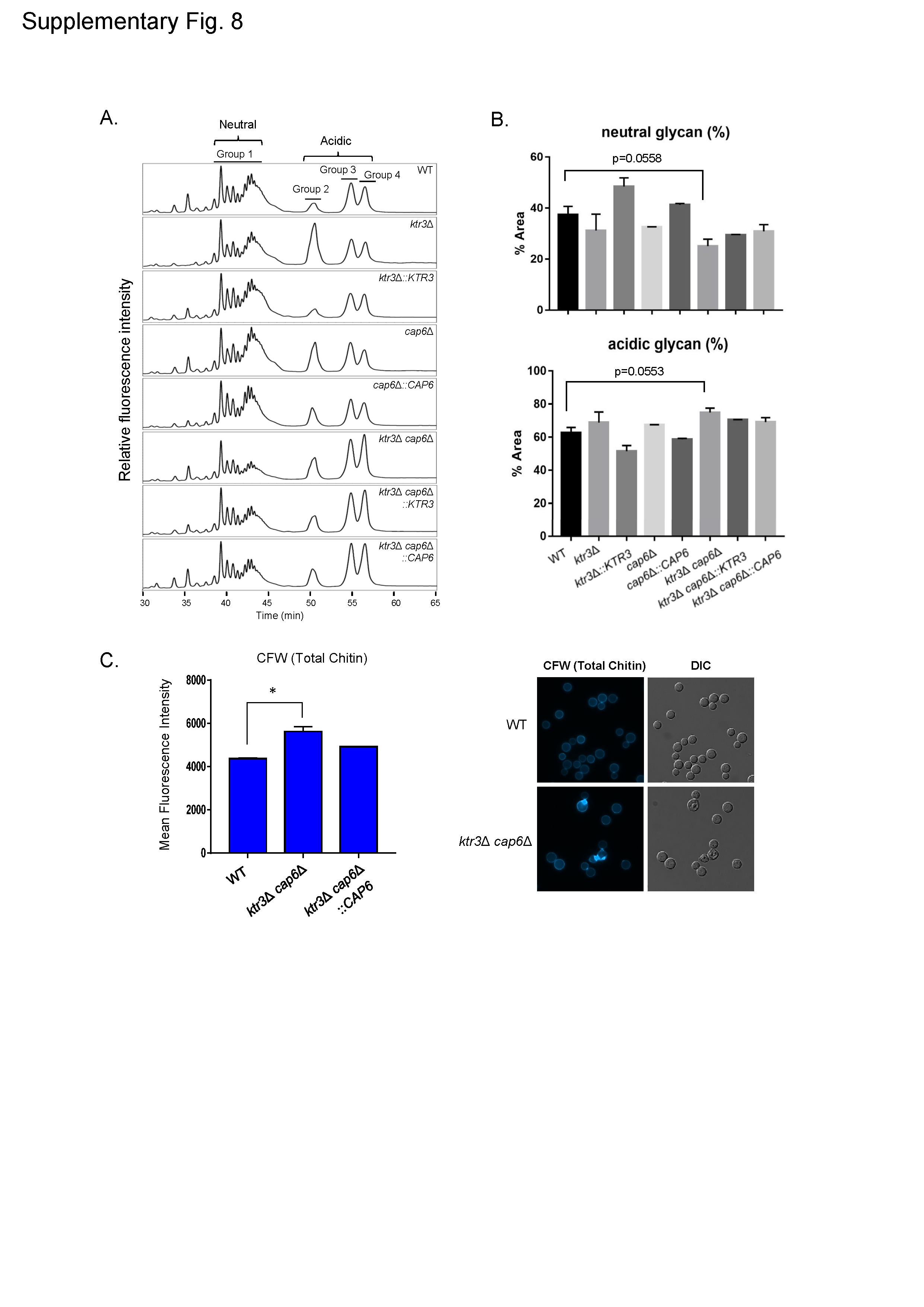

Supplement: FIG S8 [file mbio.02112-22-s0008.tif]
