# Supplementary material for: Extension of O-Linked Mannosylation in the Golgi Apparatus Is Critical for Cell Wall Integrity Signaling and Interaction with Host Cells in Cryptococcus neoformans Pathogenesis
Source: mBio. 2022 Nov 21;13(6):e02112-22. doi: 10.1128/mbio.02112-22 (PMC9765558; doi:10.1128/mbio.02112-22)
Supplement: TEXT S1 [file mbio.02112-22-s0009.docx]

**Supplemental Methods and References**

**Supplementary Methods**

**Construction of *CAP6* deletion mutant and complementation strains**

In the *C. neoformans* serotype A strain H99 (*MAT*α), genes were disrupted by biolistic transformation using double joint-PCR strategies (10). For the construction of the *CAP6* (CNAG_06016) deletion mutant strains, the DNA fragments containing the 5′- or 3′-flanking regions of ORF were PCR-amplified from H99 genomic DNA with the primer sets CN_06016D_L1/CN_06016D_L2 and CN_06016D_R1/CN_06016D_R2 (Table S1C). The 5′- and 3′-regions of the selectable marker nourseothricin acetyltransferase (NAT) were amplified with the primer sets M13Fe/NSL-2 and M13Re/NSR-2 using pNAT-STM as a template. The 5′-*CAP6-NAT* and the *NAT*- 3′-*CAP6*-fusion products were generated by overlap PCR using the primer sets CN_06016D_L1/NSL-2 and CN_06016D_R2/NSR-2, respectively. The 5′- and 3′-fragments of the *CAP6* disruption cassette was introduced into *C. neoformans* serotype A strain H99 (MATα) by biolistic transformation. Transformants were selected on YPD_NAT_, and the *CAP6* gene disruption was screened by PCR (Fig. S2A. Top). For the construction of the *KTR3* (CNAG_03832) and *CAP6* (CNAG_06016) double mutant strain, the 5′- and 3′-regions of the selectable NEO were amplified with the primer sets M13Fe/B1886 and M13Re/B1887, respectively, using pJAF1 as a template. The 5′-*CAP6-NEO* and the *NEO*-3′-*CAP6*-fusion products were generated by overlap PCR using the primer sets CN_06016D_L1/B1887 and CN_06016D_R2/B1886, respectively. The 5′- and 3′-fragments of the *CAP6* disruption cassette were introduced into *C. neoformans* *ktr3*Δ mutant background by biolistic transformation. Transformants were selected on YPD_NEO_, and gene disruption was screened by PCR (Fig. S2A, Bottom).

To construct the *CAP6*-complemented strains, *cap6*Δ*::CAP6* and *ktr3*Δ *cap6*Δ*::CAP6*, the PCR fragment containing the promoter, full-length of ORF, and terminator was obtained from the H99 genomic DNA using the primer sets CN_06016_cp_F_Xho1 and CN_06016_cp_B_EcoRV. The PCR product was cloned as a XhoⅠ/EcoRⅤ fragment into pJAFS1, generating pJAFS1-CnCAP6. For transformation of *ktr3*Δ *cap6*Δ, the *NEO* marker gene in pJAFS1-CnCAP6 was replaced with the *HYG* marker gene, from pJAF-HYG, generating pJAF(HYG)-CAP6. The resultant vectors were excised at the single EcoNⅠ site and reintegrated into the native *CAP6* promoter locus of the *cap6*Δ and *ktr3*Δ *cap6*Δ by biolistic transformation, respectively (Fig. S2B).

**Construction of a *KTR3-*complementation strain using safe haven site**

To generate the *KTR3-*complemented *ktr3*Δ *cap6*Δ mutant strain in which *KTR3* is integrated into safe have site (Fig. S2C), a DNA fragment containing *KTR3* was amplified by PCR and subcloned into pJAF-HYG containing the hygromycin resistance marker. The intergenic region between CNAG_00777 and CNAG_00778 ORFs (13) was PCR amplified from genomic DNA as two separate fragments. The 5’-flanking region of safe haven site was amplified using the primer pair, 5'frag F2 and 5'frag B2, while the 3’-flanking region of safe haven site was amplified using the primer pair, 3'frag F2 and 3'frag B2 (Table S1C). The two fragments containing the MluⅠ site were joined via overlap PCR using primers 5'frag F2 and 3'frag B2. The resulting DNA fragment was cloned into pJAFS1-CANG 03832 HYG by In-Fusion HD Cloning (Takara Bio) to generate plasmid pJAFS1-CANG 03832 HYG haven. The resultant vector was excised at the single MluⅠ site and integrated by homologous recombination into the safe haven locus of the *ktr3*Δ *cap6*Δ mutant strain by biolistic transformation. All transformants were selected on YPD supplemented with hygromycin B and confirmed by PCR screening. Integration of *KTR3* full ORF (CANG 03832) was confirmed using primers CNAG 03832 ORF F and CNAG 03832 ORF B. The integration at haven site was screened by PCR using the primers UQ2962, UQ2963, haven IN F4, and haven IN B4 (13). The primer pairs CNAG 03832 ORF F/CNAG 03832 ORF B and haven IN F4/haven IN B4 produce a 1,600 bp and 1,830 bp PCR amplicon, respectively (Fig. S2C).

**Construction of *C. neoformans* strains expressing epitope-tagged MP88**

To construct an expression vector for 6His-tagged MP88, the PCR fragment of CNAG_00776_Terminator was obtained from the H99 genomic DNA using the primers CNAG_00776_T_F_HindⅢ and CNAG_00776_T_B_ NotⅠ (Table S1C) and subcloned into pJAFS1, generating pJAFS1_CNAG_00776Ter. The DNA fragments containing the truncated ORFs of MP88 was PCR-amplified with the primers, CNAG_00776_F2_ApaⅠ, CNAG_00776_B_His_HindⅢ containing the 6His codon as C-terminal histidine tagging without GPI-anchor (Table S1C). The PCR product of truncated *MP88* ORF was digested with ApaI/HindⅢ and ligated into ApaI/HindⅢ-digested pJAFS1_CNAG_00776Ter, resulting in pJAFS1-CNAG 00776His carrying the G418 resistance marker. The vector was linearized with AvaI for targeted integration into the native locus via single homologous recombination, generating *C. neoformans* strains expressing a secretory 6His-tagged MP88 protein.

To express the MP88 protein in *ktr3*Δ *cap6*Δ double mutant strains, the NEO marker in pJAFS1-CNAG 00776His was changed to HYG marker. To obtain the region of CNAG_00776 ORF with 6HIS tagging, pJAFS1-CNAG 00776His was treated with ApaⅠ and NotⅠ. The fragment was cloned to ApaⅠ/NotⅠ treated pJAF-HYG, generating pJAF_HYG-CNAG 00776His, followed being by linearized with AvaⅠ and integrated by homologous recombination into the gene locus of the *ktr3*Δ *cap6*Δ mutant strain.

**Construction of *C. neoformans* strains expressing epitope-Wml1 and Wml2**

To construct an expression vector for Wml1 tagged with HA at its N-terminus, the PCR fragment of CNAG_01255_Terminator was obtained from the H99 genomic DNA using the primers CN1255TF_SmaⅠ and CN1255TB_XbaⅠ (Table S1C) and subcloned into pJAFS1, generating pJAFS1_CNAG_01255_Ter. The PCR fragment containing the promoter and full-length of ORF was obtained from the H99 genomic DNA using the primer, CN1255pOF_SalⅠ and CN1255pOB_SmaⅠ and subcloned into pJAFS1_CNAG_01255_Ter, generating pJAFS1-CN1255p-ORF. To obtain the 6HA fragment, the PCR fragment amplified from pHIGAZ6HA vector using the primers, EcoRⅤ-3HA_F and 3HA-EcoRⅤ_B. The 6HA PCR fragment was treated with EcoRⅤ and inserted at the EcoRⅤ site (blunt end) in the N-terminal region of CNAG_01255 ORF, generating pJAFS1-CN1255NHA.

To construct an expression vector for Wml2 tagged with HA at its C-terminus, the PCR fragment of CNAG_03328_Terminator was obtained from the H99 genomic DNA using the primers CN3328TF_SmaⅠ and CN3328TB_XbaⅠ (Table S1C) and subcloned into pJAFS1, generating pJAFS1_CNAG_03328_Ter. The PCR fragment containing the promoter and full-length of ORF was obtained from the H99 genomic DNA using the primer, CN3328pOF_KpnⅠ and CN3328pOB_SmaⅠ and subcloned into pJAFS1_CNAG_03328_Ter, generating pJAFS1-CN3328p-ORF. To obtain the 6HA fragment, the PCR fragment amplified from pHIGAZ6HA vector using the primer, EcoRⅤ-3HA_F and 3HA-EcoRⅤ_B. The 6HA PCR fragment was treated with EcoRⅤ and inserted at the StuⅠ site (blunt end) in the C-terminal region of CNAG_03328 ORF, generating pJAFS1-CN3328CHA.

To express the Wml1 and Wml2 proteins in the WT, *ktr3*Δ, or *cap6*Δ mutant strains, pJAFS1-CN1255NHA and pJAFS1-CN3328CHA vectors were linearized with CsilⅠ and SalⅠ, respectively, and integrated by homologous recombination into the gene locus of the strains.

To express the Wml1 protein in the *ktr3*Δ *cap6*Δ double mutant strain, the NEO marker in pJAFS1-CN1255NHA was changed to HYG. By exchanging the PsiⅠ/Bsu36Ⅰ DNA fragment for NEO with the PsiⅠ/Bsu36Ⅰ DNA fragment of HYG marker, generating pJAF-CN1255NHA-HYG. After being linearized with CsiⅠ, pJAF-CN1255NHA-HYG was integrated by homologous recombination into the gene locus of the *ktr3*Δ *cap6*Δ mutant strain. To express the Wml2 protein in the *ktr3*Δ *cap6*Δ strain, the region of full-length of CNAG_03328 ORF containing the promoter was obtained from the pJAFS1-CN3328CHA vector by PCR using NotⅠ_CN3328_F and CN3328_EcoRV_B primers. The PCR fragment treated with NotⅠ/EcoRⅤ was cloned to pJAF-HYG, generating pJAF-CN3328CHA-HYG, followed being by linearized with SalⅠ and integrated by homologous recombination into the gene locus of the *ktr3*Δ *cap6*Δ strain.

**Construction of *C. neoformans* strains expressing FLAG-tagged Mpk1**

To construct expression vector for FLAG-tagged Mpk1, the PCR fragment of CNAG_04514_Terminator was obtained from the H99 genomic DNA using the primers SalⅠ_MPK1_Ter and MPK1_Ter_kpnⅠ (Table S1C) and subcloned into pJAFS1, generating pJAFS1_CNAG_04514_Ter. The DNA fragments containing the truncated ORFs of *MPK1* was PCR-amplified with the primer sets, NotⅠ_MPK1ORF and MPK1ORF(FLAG)_SalⅠ, containing the FLAG codon as C-terminal FLAG tagging (Table S1C). The PCR product of truncated *MPK1* ORF was digested with NotⅠ/SalⅠ and ligated into NotⅠ/SalⅠ-digested pJAFS1_CNAG_04514Ter, resulting in pJAFS1-CN4514(FLAG) carrying the G418 resistance marker. The pJAFS1-CN4514(FLAG) vector was linearized with CsiI, for targeted integration into the native locus via single homologous recombination, generating the *C. neoformans* strains expressing Mpk1-FALG.

**Construction of cell wall stress sensors mutants and complementation strains**

For the construction of the *WML1* (CNAG_01255) or *WML2* (CNAG_03328) null mutant strain, DNA fragments containing the 5′- or 3′-flanking regions of ORF were PCR-amplified from H99 genomic DNA with the primer sets CN_01255D_L1/CN_01255D_L2 and CN_01255D_R1/CN_01255D_R2, CN_03328D_L1/CN_03328D_L2 and CN_03328D_R1/CN_03328D_R2, respectively (Table S1C). The 5′- and 3′-regions of the selectable marker nourseothricin acetyltransferase (NAT) were amplified with the primer sets M13Fe/NSL-2 and M13Re/NSR-2 using pNAT-STM as a template. *WML1-NAT* fusion products of 5′- and 3′-flanking regions were generated by overlap PCR using the primer sets CN_01255D_L1/NSL-2 and CN_01255D_R2/NSR-2, respectively. The 5′- and 3′-*WML2-NAT* fusion products were generated by overlap PCR using the primer sets CN_03328D_L1/NSL-2 and CN_03328D_R2/NSR-2, respectively. The 5′- and 3′-fragments of the *WML1* or *WML2* disruption cassette were introduced into *C. neoformans*, transformants were selected on YPD_NAT_, and gene disruption was screened by PCR. For the construction of the *WML1* (CNAG_01255) and *WML2* (CNAG_03328) double mutant strain, the 5′- and 3′-regions of NEO were amplified with the primer sets M13Fe/B1886 and M13Re/B1887, respectively, using pJAF1 as a template. The 5′- and 3′*WML2-NEO* fusion products were generated by overlap PCR using the primer sets CN_03328D_L1/B1887 and CN_03328D_R2/B1886, respectively. The 5′- and 3′-fragments of the *WML2* disruption cassette were introduced into *C. neoformans* *wml1*Δ mutant background by biolistic transformation. To generate *WML2-*complemented strains, a DNA fragment containing the *WML2* gene was amplified by PCR and subcloned into pJAF-HYG containing the HYG marker. The resultant vector was excised at the single MluⅠ site and reintegrated into the native *WML2* promoter locus of the *wml1*Δ *wml2*Δ strain by biolistic transformation (Fig. S4).

**Partial fractionation and western blot analysis of Wml1 and Wml2**

To carry out partial fractionation of Wml1 and Wml2 for western blot analysis*, C. neoformans* cells were inoculated at an initial OD_600_ of 0.5 and cultured in 50 ml YPD at 30°C for 8 h. Cells were harvested, washed with TNE buffer (50 mM Tris-HCl [pH 7.5], 150 mM NaCl, 5 mM EDTA [pH 8]) and resuspended in lysis buffer (50 mM Tris-HCl [pH 7.5], 150 mM NaCl, 5 mM EDTA [pH 8], 1 mM phenylmethyl sulfonyl fluoride [PMSF], and 1x protease inhibitor cocktail [PIC]). The cells were divided into two tubes to extract total proteins and to fractionate soluble/insoluble proteins. The same volume of glass bead (425–600 μm in diameter, Sigma) was added and the cells were disrupted 4 times for 15 seconds at 5,000 rpm using a Precellys^®^ 24 Tissue Homogenizer (Bertin Technologies). To extract the total proteins, 5x sample loading buffer (62.5 mM Tris-HCl [pH 6.8], 2.5 % SDS, 0.002 % Bromophenol Blue, 5% β-mercaptoethanol, 10 % glycerol) was added to cell lysates and boiled for 10 min. Cell debris and glass beads were removed by centrifugation for 10 min at 16,000 g. To obtain the soluble proteins fraction, cell lysates were centrifuged for 10 min at 16,000 g and supernatant was saved, added 5x sample loading buffer and boiled for 10 min. To obtain the insoluble protein fraction, the 1x sample loading buffer was added into the remaining cell pellets and boiled for 10 min. Protein lysates adjusted to the same concentration were separated by SDS-PAGE electrophoresis and transferred to PVDF membranes. The HA-tagged Wml1 and Wml2 proteins were analyzed by western blotting with anti-HA High Affinity Rat monoclonal antibody (Roche).

**Western blot analysis of Mpk1 and Hog1 phosphorylation**

To detect the expression of phospho-Mpk1, *C. neoformans* cells were grown to an OD_600_ of 1.0 in YPD medium at 30°C, and then one-half of the cells was harvested, while the second half was treated with tunicamycin (TM, 5 μg/ml) at 30°C for 2 h. The cells were resuspended in a phosphatase inhibitor lysis buffer (50 mM Tris–HCl [pH 7.5], 10 mM sodium orthovanadate, 5 mM sodium pyrophosphate, 50 mM sodium fluoride, 1% [wt/vol] sodium deoxycholate, 0.1% [wt/vol] SDS, 1% [vol/vol] Triton X-100, 1 mM phenylmethylsulfonyl fluoride [PMSF], and 1x protease inhibitor cocktail [PIC]) with glass beads (425–600 μm in diameter, Sigma) and disrupted using Precellys^®^ 24 Tissue Homogenizer (Bertin Technologies) 4 times for 15 sec (5,000 rpm). The soluble proteins were analyzed by western blotting with phospho-p44/42-MAPK antibody (Cell Signaling Technology) for Mpk1-P protein and anti-Hog1 antibody (Santa Cruz biotechnology) for the Hog1 protein as a loading control.

To monitor Hog1 phosphorylation patterns, the *C. neoformans* strains were synchronized from OD_600_ 0.2 to an OD_600_ 0.8 in YPD medium at 30°C, and a portion of the cell culture was harvested, while the remaining culture was mixed with equal volume of YPD medium containing 2 M NaCl (final 1 M NaCl) and further incubated for the indicated times (0, 15, 30, 60 min). After the indicated timepoint, the cells were harvested by centrifugation (3000 rpm, 1977 rcf) at 4°C for 5 min and immediately frozen with liquid nitrogen. Frozen samples were resuspended in the lysis buffer with silica beads and disrupted using a bead beater (FastPrep-24^TM^ 5G, MPBio). The soluble proteins were analyzed by western blotting using phospho-p38-MAPK antibody (Cell Signaling Technology, #4511) for monitoring Hog1 phosphorylation and customized anti-cryptococcal Hog1 polyclonal antibody (GW Vitek, South Korea) for measuring the total Hog1 proteins as a loading control.

**Preparation of RNA, RNA-sequencing, and qRT-PCR analysis**

*C. neoformans* WT and mutant cells at OD_600_ of 0.15 were cultured in 50 ml of YPD broth at 30°C in a shaking incubator until the OD_600_ reached 0.8, the early phase of exponential growth. The cells were then treated with 5 μg/ml tunicamycin (TM) and further incubated at 30°C in a shaking incubator for 1 h. The cell pellets were washed with diethyl pyrocarbonate (DEPC)-treated water twice, immediately frozen in liquid nitrogen, and disrupted thoroughly using a mortar and pestle. Total RNA was extracted using the RNeasy Mini Kit (Qiagen) and subjected to RNA formaldehyde-agarose gel electrophoresis.

For RNA-sequencing, the NEBNext Ultra Ⅱ Directional RNA-Seq Kit (NEW ENGLAND BioLabs) was used to prepare libraries. The mRNA, isolated by using the Poly(A) RNA Selection Kit (LEXOGEN), was used for the cDNA synthesis. The Illumina indexes 1–12 were used to perform indexing and enrichment step was carried out. TapeStation HS D1000 Screen Tape (Agilent Technologies) was used to check libraries for evaluating the mean fragment size. Quantification was conducted on a StepOne Real-Time PCR System (Life Technologies) using the library quantification kit, and NovaSeq 6000 (Illumina) was used for high-throughput sequencing as paired-end 100 sequencing. The raw sequencing data was checked for quality control using FastQC. FASTX_Trimmer and BBMap were used to remove adapter and low-quality reads (<Q20), then TopHat was used to map the trimmed reads with reference genome. Gene expression levels were estimated using FPKM (fragments per kb per million reads) values, which normalized using EdgeR within R. RNA-seq analysis was conducted using the ExDEGA (ebiogen). Raw RNA-sequencing data have been submitted to the NCBI GEO database under accession no. GSE198875.

To validate the RNA-sequencing, cDNA was synthesized using a SuperiorScript III Master Mix (Enzynomics). Quantitative real-time PCR (qRT-PCR) was carried out with CFX96 Optical Module (Biorad) using TB Green Premix Ex Taq (Takara). The Gene expression values were calculated by the 2^−ΔΔCT^ method with GAPDH as a control, and all PCR reactions were analyzed in duplicate the primers listed in Table S1C.

**Supplementary References**

1. Perfect JR, Ketabchi N, Cox GM, Ingram CW, Beiser CL. 1993. Karyotyping of *Cryptococcus neoformans* as an epidemiological tool. *J Clin Microbiol* 31:3305-3309.2.

2. Lee DJ, Bahn YS, Kim HJ, Chung SY, Kang HA. 2015. Unraveling the novel structure and biosynthetic pathway of *O*-linked glycans in the Golgi apparatus of the human pathogenic yeast *Cryptococcus neoformans*. *J Biol Chem* 290:1861–1873.

3. Bahn YS, Hicks JK, Giles SS, Cox GM, Heitman J. 2004. Adenylyl cyclase-associated protein Aca1 regulates virulence and differentiation of *Cryptococcus neoformans* via the cyclic AMP-protein kinase A cascade. *Eukaryot Cell* 3:1476-1491.

4. Kojima K, Bahn YS, Heitman J. 2006. Calcineurin, Mpk1 and Hog1 MAPK pathways independently control fludioxonil antifungal sensitivity in *Cryptococcus neoformans*. *Microbiology* 152:591-604.

5. Lee KT, Hong J, Lee DG, Lee M, Cha S, Lim YG, Jung KW, Hwangbo A, Lee Y, Yu SJ, Chen YL, Lee JS, Cheong E, Bahn YS. 2020. Fungal kinases and transcription factors regulating brain infection in *Cryptococcus neoformans*. *Nat Commun* 11:1521.

6. Jin JH, Lee KT, Hong J, Lee D, Jang EH, Kim JY, Lee Y, Lee SH, So YS, Jung KW, Lee DG, Jeong E, Lee M, Jang YB, Choi Y, Lee MH, Kim JS, Yu SR, Choi JT, La JW, Choi H, Kim SW, Seo KJ, Lee Y, Thak EJ, Choi J, Averette AF, Lee YH, Heitman J, Kang HA, Cheong E, Bahn YS. 2020. Genome-wide functional analysis of phosphatases in the pathogenic fungus *Cryptococcus neoformans*. *Nat Commun* 11:4212.

7. Bahn YS, Kojima K, Cox GM, Heitman J. 2005. Specialization of the HOG pathway and its impact on differentiation and virulence of *Cryptococcus neoformans*. *Mol Biol Cell* 16:2285-2300.

# 8. So YS, Jang J, Park G, Xu J, Olszewski MA, Bahn YS. 2018. Sho1 and Msb2 Play Complementary but Distinct Roles in Stress Responses, Sexual Differentiation, and Pathogenicity of *Cryptococcus neoformans*. *Front Microbiol* 9:2958.

9. Cheon SA, Jung KW, Chen YL, Heitman J, Bahn YS, Kang HA. 2011. Unique evolution of the UPR pathway with a novel bZIP transcription factor, Hxl1, for controlling pathogenicity of *Cryptococcus neoformans*. *PLoS Pathog* 7:e1002177.

10. Kim MS, Kim SY, Yoon JK, Lee YW, Bahn YS. 2009. An efficient gene-disruption method in *Cryptococcus neoformans* by double-joint PCR with *NAT*-split markers. *Biochem Biophys Res Commun* 390:983-988.

11. Hua J, Meyer JD, Lodge JK. 2000. Development of positive selectable markers for the fungal pathogen *Cryptococcus neoformans*. *Clin Diagn Lab Immunol* 7:125-128.

12. Kim H, Moon HY, Lee DJ, Cheon SA, Yoo SJ, Park JN, Agaphonov MO, Oh DB, Kwon O, Kang HA. 2013. Functional and molecular characterization of novel *Hansenula polymorpha* genes, *HpPMT5* and *HpPMT6*, encoding protein *O*-mannosyltransferases. *Fungal Genet. Biol* 58-59;10–24.

13. Arras SDM, Chitty JL, Blake KL, Schulz BL, Fraser JA. 2015. A Genomic Safe Haven for Mutant Complementation in *Cryptococcus neoformans*. *PLoS One* 10:e0122916.
