# Supplementary material for: Extension of O-Linked Mannosylation in the Golgi Apparatus Is Critical for Cell Wall Integrity Signaling and Interaction with Host Cells in Cryptococcus neoformans Pathogenesis
Source: mBio. 2022 Nov 21;13(6):e02112-22. doi: 10.1128/mbio.02112-22 (PMC9765558; doi:10.1128/mbio.02112-22)
Supplement: TABLE S1 [file mbio.02112-22-s0010.docx]

**Table S1A. Strains used in this study.**

| **Strain** | **Genotype** | **Parent** | **Reference** |
| --- | --- | --- | --- |
| *C*. *neoformans* H99 | *MAT*α (serotype A) |  | (1) |
| *ktr3*Δ | *MAT*α Cn03832*::NAT#*159 | H99 | (2) |
| *ktr3*Δ*::KTR3* | *MAT*α Cn03832*::NAT#*159 Cn03832-*NEO* | *ktr3*Δ | (2) |
| *cap6*Δ | *MAT*α Cn06016*::NAT#*159 | H99 | This study |
| *cap6*Δ*::CAP6* | *MAT*α Cn06016*::NAT#*159 Cn06016-*NEO* | *cap6*Δ | This study |
| *ktr3*Δ *cap6*Δ | *MAT*α Cn03832*::NAT#*159 Cn06016::*NEO* | *ktr3*Δ | This study |
| *ktr3*Δ *cap6*Δ*::CAP6* | *MAT*α Cn03832*::NAT#*159 Cn06016::*NEO* Cn6016*-HYG* | *ktr3*Δ *cap6*Δ | This study |
| *ktr3*Δ *cap6*Δ*::KTR3* | *MAT*α Cn03832*::NAT#*159 Cn06016::*NEO* Cn3832*-HYG* | *ktr3*Δ *cap6*Δ | This study |
| *wml1*Δ | *MAT*α Cn01255*::NAT#159* | H99 | This study |
| *wml2*Δ | *MAT*α Cn03328*::NAT#159* | H99 | This study |
| *wml1*Δ *wml2*Δ | *MAT*α Cn01255*::NAT#*159 Cn03328*::NEO* | *wml1*Δ | This study |
| *wml1*Δ *wml2*Δ*::WML2* | *MAT*α Cn01255*::NAT#*159 Cn03328*::NEO* Cn03328*-HYG* | *wml1*Δ *wml2*Δ | This study |
| H99*/*Wml1_NHA | *MAT*α Cn01255-6HA | H99 | This study |
| *ktr3*Δ*/*Wml1_NHA | *MAT*α Cn03832*::NAT#*159 Cn01255-6HA | *ktr3*Δ | This study |
| *cap6*Δ*/*Wml1_NHA | *MAT*α Cn06016*::NAT#*159 Cn01255-6HA | *cap6*Δ | This study |
| *ktr3*Δ *cap6*Δ*/*Wml1_NHA | *MAT*α Cn03832*::NAT#*159 Cn06016*::NEO* Cn01255*-*6HA | *ktr3*Δ *cap6*Δ | This study |
| H99*/* Wml2_CHA | *MAT*α Cn03328-6HA | H99 | This study |
| *ktr3*Δ*/*Wml2_CHA | *MAT*α Cn03832*::NAT#*159 Cn03328*-*6HA | *ktr3*Δ | This study |
| *cap6*Δ*/*Wml2*_*CHA | *MAT*α Cn06016*::NAT#*159 Cn03328*-*6HA | *cap6*Δ | This study |
| *ktr3*Δ *cap6*Δ*/* Wml2_CHA | *MAT*α Cn03832*::NAT#*159 Cn06016*::NEO* Cn03328-6HA | *ktr3*Δ *cap6*Δ | This study |
| MP88(H) | *MAT*α Cn00776(H)*::NEO* | H99 | This study |
| *alg3*Δ*/* MP88(H) | *MAT*α Cn05142*::NAT#*159 Cn00776(H)*::NEO* | *alg3*Δ | This study |
| *ktr3*Δ*/* MP88(H) | *MAT*α *Cn03832::NAT#*159 Cn00776(H)*::NEO* | *ktr3*Δ | This study |
| *cap6*Δ*/* MP88(H) | *MAT*α *Cn06016::NAT#*159 Cn00776(H)*::NEO* | *cap6*Δ | This study |
| *ktr3*Δ *cap6*Δ*/* MP88(H) | *MAT*α Cn03832*::NAT#*159 Cn06016*::NEO* Cn00776(H)*::HYG* | *ktr3*Δ *cap6*Δ | This study |
| *cac1*Δ | *MAT*α *cac1*Δ*::NAT*#159 | H99 | (3) |
| H99*/*Mpk1-FLAG | *MAT*α Cn04514(H)*::NEO* | H99 | This study |
| *mpk1*Δ (*KK3*) | *MAT*α *mpk1*Δ*::NAT*#150 | H99 | (4) |
| *cap59*Δ | *MAT*α Cn00721::*HYB* | H99 | This study |
| *ktr3*Δ *cap59*Δ | *MAT*α Cn03832*::NAT#*159 Cn00721::*HYB* | *ktr3*Δ | This study |
| *ktr3*Δ*::KTR3 cap59*Δ | *MAT*α Cn03832*::NAT#*159 Cn03832-*NEO* Cn00721::*HYB* | *ktr3*Δ*::KTR3* | This study |
| *cap6*Δ *cap59*Δ | *MAT*α Cn06016*::NAT#*159 Cn00721::*HYB* | *cap6*Δ | This study |
| *cap6*Δ*::CAP6 cap59*Δ | *MAT*α Cn06016*::NAT#*159 Cn06016-*NEO* Cn00721::*HYB* | *cap6*Δ*::CAP6* | This study |
| *ktr3*Δ *cap6*Δ *cap59*Δ | *MAT*α Cn03832*::NAT#*159 Cn06016::*NEO* Cn00721::*HYB* | *ktr3*Δ *cap6*Δ | This study |
| *alg3*Δ *cap59*Δ | *MAT*α Cn05142*::NAT#*159 Cn00721::*HYB* | *alg3*Δ | This study |
| *mpr1*Δ (YSB5492) | *MAT*α *mpr1*Δ*::NAT* | H99 | (5) |
| *ssu72*Δ (YSB4242) | *MAT*α *ssu72*Δ*::NAT*#119 | H99 | (6) |
| *gda1*Δ (YSB4750) | *MAT*α *gda1*Δ*::NAT*#6 | H99 | (6) |
| *hog1*Δ (YSB64) | *MAT*α *hog1*Δ*::NAT*#177 | H99 | (7) |
| *msb2*Δ (YSB3191) | *MAT*α *msb2*Δ*::NAT*#150 | H99 | (8) |
| *hxl1*Δ (YSB723) | *MAT*α *hxl1*Δ*::NAT*#229 | H99 | (9) |

*Each *NAT-STM#* indicates the Nat^r^ marker with a unique signature tag

**Table S1B. Plasmids used in this study.**

| **Plasmid** | **Description** | **Reference** |
| --- | --- | --- |
| pNAT-STM#159 | NAT-resistant marker vector for gene disruption | (10) |
| pJAF15 | pJAF-based vector containing hygromycin B marker | (11) |
| pJAFS1 | NEO-resistant marker vector | (9) |
| pHIGAZ-6HA | pUC-based vector containing a zeocin marker and six copies of hemagglutinin (HA) tag | (12) |
| pJAFS1-CnCAP6 | pJAFS-based expression vector containing CAP6 fragment with the neomycin marker | This study |
| pJAF (HYG)-CAP6 | pJAF-based expression vector containing CAP6 fragment with the hygromycin B marker | This study |
| pJAFS1-CNAG 00776His | pJAFS1-based expression vector for six histidine-tagged MP88 with the neomycin marker | This study |
| pJAF_HYG-CNAG 00776His | pJAFS1-based expression vector for six histidine-tagged MP88 with the hygromycin marker | This study |
| pJAFS1-CN1255NHA | pJAFS1-based expression vector for six hemagglutinin-tagged Wml1 | This study |
| pJAFS1-CN3328CHA | pJAFS1-based expression vector for six hemagglutinin-tagged Wml2 | This study |
| pJAF1-CN1255NHA (HYG) | pJAF1-based expression vector for six hemagglutinin-tagged Wml1 | This study |
| pJAF1-CN3328CHA (HYG) | pJAF1-based expression vector for six hemagglutinin-tagged Wml2 | This study |
| pJAFS1-Mpk1(FLAG) | pJAFS1-based expression vector for FLAG-tagged Mpk1 | This study |
| pJAF-CN3328-HYG | pJAFS1 containing the Wml2 ORF | This study |
| pJAFS1_CNAG03832-HYG haven | pJAFS1 containing the Ktr3 ORF | This study |

**Table S1C. Oligonucleotides used in this study.**

| **Name** | **Sequence (5′-3′)** | **Purpose** |
| --- | --- | --- |
| M13Fe | GTAAAACGACGGCCAGTGAGC | Screening primer for dominant selectable marker gene (*NAT/NEO/HYG*) |
| NSL-2 | AACTCCGTCGCGAGCCCCATCAAC | 5′-Region of *NAT* split marker |
| M13Re | CAGGAAACAGCTATGACCATG | Screening primer for dominant selectable marker gene (*NAT/NEO/HYG*) |
| NSR-2 | AAGGTGTTCCCCGACGACGAATCG | 3′-Region of *NAT* split marker |
| B1886 | TGGAAGAGATGGATGTGC | Primer for 5’-split-region of *NEO* |
| B1887 | ATTGTCTGTTGTGCCCAG | Primer for 3’-split-region of *NEO* |
| CN_6016D_L1 | GTAATGACGAGTGCGAGA | *CAP6* disruption cassette |
| CN_6016D_L2 | GCTCACTGGCCGTCGTTTTACATCCGATGGGCTATACAG | *CAP6* disruption cassette |
| CN_6016D_R1 | CATGGTCATAGCTGTTTCCTGCTTCCGCTTCTGAGACTA | *CAP6* disruption cassette |
| CN_6016D_R2 | GCAAGGTACTCTGACATG | *CAP6* disruption cassette |
| CN_6016D_Sc_F | GTGTCCGTGCTTGTAATG | Confirmation of *CAP6* disruption |
| CnD-ACTsqB(B79y) | TGTGGATGCTGGCGGAGGATA | Confirmation of *CAP6* disruption |
| CN_6016D_Sc_F2 | GTCCCATCTGATGTTTCG | Confirmation of CAP6 integration |
| CN_06016_cp_F_XhoⅠ | CCACTCGAGGTGTCCGTGCTTGTAATG | Integration of *CAP6* |
| CN_06016_cp_B_EcoRV | TACGATATCTCGCACAACGACTTAGCA | Integration of *CAP6* |
| CN_6016_orf_F | GGTCAGAGTGCTACTGAT | Confirmation of CAP6 |
| CN_6016_orf_B | AACTCACCAGCAACCTGA | Confirmation of CAP6 |
| CN_01255D_L1 | caaagcaccaagccatac | *WML1* disruption cassette |
| CN_01255D_L2 | GCTCACTGGCCGTCGTTTTACggtggagggattgtaaag | *WML1* disruption cassette |
| CN_01255D_R1 | CATGGTCATAGCTGTTTCCTGtgtacccaggagaattgc | *WML1* disruption cassette |
| CN_01255D_R2 | tatgcgctgagcaaggaa | *WML1* disruption cassette |
| CNWML1D-confirmF | tcgacttctatctcgtcc | Confirmation of *WML1* disruption |
| CNWML1D-confirmB | ccatactcgtatccatgc | Confirmation of *WML1* disruption |
| CN_03328D_L1 | agcttagtgggtgactca | *WML2* disruption cassette |
| CN_03328D_L2 | GCTCACTGGCCGTCGTTTTACgatgatgagtgactgacc | *WML2* disruption cassette |
| CN_03328D_R1 | CATGGTCATAGCTGTTTCCTGcgccatcttggctttgat | *WML2* disruption cassette |
| CN_03328D_R2 | aaaggctaatcggcccat | *WML2* disruption cassette |
| CNWML2D-confrimF | ctcttgctcatgtcgatc | Confirmation of *WML2* disruption |
| CNWML2D-confrimB | aggtagatgaagcggtag | Confirmation of *WML2* disruption |
| Not1CnWML2D_F | aagcggccgcccggtttggggttgttta | Integration of *WML2* |
| CnWML2D_B | accctcactaaagggaac | Integration of *WML2* |
| CN1255pOF_SalⅠ | ccacgtcgaccctagatggaccta | Amplification of *WML1* promoter-ORF |
| CN1255pOB_SmaⅠ | GACCCGGGCTAATACCGGAAATTGGCAGC | Amplification of *WML*1 promoter-ORF |
| CN1255TF_SmaⅠ | gacccgggactgtcgtttcatcttaggct | Amplification of *WML1* terminator |
| CN1255TB_XbaⅠ | AGTTCTAGATACTATACGCCCGACAAC | Amplification of *WML1* terminator |
| CN3328pOF_KpnⅠ | ctcggtaccccggtttggggttgtttatc | Amplification of *WML2* promoter-ORF |
| CN3328pOB_SmaⅠ | GACCCGGGTTAAGGCCTCCTGTAGTCATGAGGATTGG | Amplification of *WML2* promoter-ORF |
| CN3328TF_SmaⅠ | gacccggggaatagtctggggcacgt | Amplification of *WML2* terminator |
| CN3328TB_XbaⅠ | AGTTCTAGATATCGTCGAGTACGCAAG | Amplification of *WML2* terminator |
| Cn1255t_IdenB | atggcctatctattgcgg | Confirmation of HA-tagged *WML1* |
| Cn3328p_IdenF | catgatgctgttacacgc | Confirmation of HA-tagged *WML2* |
| EcoRV-3HA_F | ccgatatctacccatacgatgttcctg | Amplification of 6HA |
| 3HA- EcoRV_B | ccgatatcagcgtaatctggaacgtc | Amplification of 6HA |
| NotⅠ_CN3328_F | GGGCGGCCGCccggtttggggttgttta | Amplification of *WML2* promoter-ORF |
| CN3328_EcoRV _B | GGGATATCttaaggaggagcgtaatct | Amplification of *WML2* promoter-ORF |
| CNAG_00776_B_His_ HindⅢ | gtcAAGCTTTTAGTGGTGGTGGTGGTGGTGAGAAGTAGAGCTAGAGCT | Amplification of His-tagged MP88 ORF |
| CNAG_00776_F2_ ApaⅠ | ataGGGCCCgatctccaaggttgctgt | Amplification of His-tagged MP88 ORF |
| CNAG_00776_T_F_ HindⅢ | gtcAAGCTTccgagctattgatgttttcgacg | Amplification of *MP88* terminator |
| CNAG_00776_T_B_ NotⅠ | atgcggccgcTTGGACGGATGACGAAGT | Amplification of *MP88* terminator |
| CNAG_00776_sq_O_F | acagcataccttgccgaa | Confirmation of His-tagged *MP88* |
| SalⅠ_MPK1_Ter | GCGCGTCGACAATCATATTTGTCGAGTCTGTAAC | Amplification of *MPK1* terminator |
| MPK1_Ter_kpnⅠ | GCGCGGTACCCGCACGGCGGAGGGA | Amplification of *MPK1* terminator |
| NotⅠ_MPK1ORF | AGAGGCGGCCGCTTCCAGACGCCCAACAACG | Amplification of *MPK1* ORF |
| MPK1ORF(FLAG)_SalⅠ | AGAGGTCGACCTACTTGTCGTCATCGTCTTTGTAGTCTGATAATTTCTGCCTCTCCAAC | Amplification of *MPK1* ORF |
| MPK1inte_F | CCCTCTTGTCTTGATCACTC | Confirmation of FLAG-tagged *MPK1* |
| MPK1inte_B2 | CTACTTGTCGTCATCGTC | Confirmation of FLAG-tagged *MPK1* |
